# From Tracks to Hotspots: Particle-Dependent Radiation Energy Deposition in MAPbI_3_ Perovskite

**DOI:** 10.3390/nano16130803

**Published:** 2026-06-29

**Authors:** Ivan E. Novoselov, Zhi Xing, Huiliang Sun, Ivan S. Zhidkov

**Affiliations:** 1 Institute of Physics and Technology, Ural Federal University, Mira 19 Street, Yekaterinburg 620062, Russia; 2 Federal Research Center of Problems of Chemical Physics and Medicinal Chemistry of the Russian Academy of Sciences, Semenov Av., 1, Chernogolovka 142432, Russia; 3 School of Chemistry and Chemical Engineering, Gannan Normal University, Ganzhou 341000, China; 4 Guangdong Engineering Technology Research Center for Photoelectric Sensing Materials & Devices, Guangzhou Key Laboratory of Sensing Materials & Devices, Center for Advanced Analytical Science, School of Chemistry and Chemical Engineering, Guangzhou University, Guangzhou 510006, China; 5 M.N. Mikheev Institute of Metal Physics of the Ural Branch of the Russian Academy of Sciences, S. Kovalevskoi 18 Street, Yekaterinburg 620108, Russia

**Keywords:** hybrid halide perovskites, Geant4, radiation interaction, radiation detectors, particle irradiation

## Abstract

Geant4 (version 11.3.2) simulations were used to study particle-dependent radiation interaction in MAPbI_3_ under electron, photon, and neutron irradiation. The analysis focused on spatial distributions of interaction events, released energy, secondary-particle generation, and process-specific contributions. A 1 mm single-layer MAPbI_3_ target was used to identify the intrinsic material response, while multilayer MAPbI_3_ containing detector geometries were considered to assess device-like effects. Electrons produced extended charged particle tracks governed by direct energy loss and secondary-electron cascades. Photons showed weak direct energy deposition, with the response mainly controlled by secondary electrons generated in discrete electromagnetic interactions. Neutrons produced sparse but locally intense energy-release patterns dominated by recoil particles and nuclear-reaction products. The results show that total released energy alone is insufficient to describe radiation response in MAPbI_3_; spatial morphology and the balance between primary and secondary contributions are essential for interpreting both detector operation and possible radiation-induced degradation.

## 1. Introduction

Hybrid halide perovskites have attracted major attention over the past decade because of their remarkable optoelectronic properties [1,2], compositional flexibility [3,4], and relatively accessible fabrication [5,6,7,8]. Among them, methylammonium lead iodide (MAPbI_3_) remains one of the most widely studied model materials [9,10,11] and continues to serve as a representative system for understanding the broader behavior of lead-halide perovskites. In the present work, MAPbI_3_ is used as a model compound for analyzing radiation transport in lead iodide perovskites of the APbI_3_ type. This choice is reasonable because the interaction of photons, electrons, and neutrons with these materials is strongly influenced by the Pb and I sublattice and by the effective atomic number of the absorber. Variation in the A-site cation is not expected to change the general class of radiation interaction mechanisms. However, it can affect quantitative characteristics, including the number and energy of generated secondary particles, the deposited energy distribution, and especially the chemical and radiation stability of a particular composition. Their composition includes relatively heavy elements, which can enhance interaction with ionizing radiation, while their broader material family also offers substantial tunability in composition and structure. Although these materials are primarily associated with photovoltaics and other optoelectronic applications [7,12], their interaction with ionizing radiation is also of considerable interest [13,14,15,16]. This interest is motivated not only by the need to evaluate material stability under irradiation [17,18,19], but also by the possibility of using perovskite-based systems in radiation-related technologies.

From an application perspective, this problem appears in several different contexts. One of them is operation in environments with elevated radiation levels, including near-space and space conditions, and other scenarios in which electronic and optoelectronic components are exposed to energetic particles and rays. Another important context is the use of semiconducting materials in radiation detection, monitoring, and imaging [20,21,22,23,24]. Numerical studies of proton irradiation have also shown that the response of halide perovskites depends on composition, particle energy, and the redistribution of energy between ionization, displacement, and heating channels [25]. In such cases, the material is not merely required to survive irradiation; rather, its interaction with incoming particles becomes a functional part of the device response. This is relevant to X-ray [21,26] and gamma-ray [22,26] detection, imaging-oriented structures [21,27], and, more broadly, to systems intended for radiation sensing in scientific [22,28,29], environmental [15,30], or medical [21,27] settings. Even when a specific device architecture is not modeled directly, understanding the underlying radiation–matter interaction at the material level is a necessary first step.

However, the relevance of halide perovskites to radiation-related applications cannot be assessed solely from their composition or from general expectations of strong radiation interaction. What matters in practice is not only whether the material interacts with incident radiation, but also how the energy is transferred, how it is distributed within the target volume, and which physical processes dominate under different irradiation conditions. These aspects are especially important because different classes of incident particles interact with matter through fundamentally different mechanisms and may therefore produce distinct patterns of local energy deposition.

This is particularly evident when comparing electrons, photons, and neutrons in the same material. Electrons typically undergo repeated inelastic interactions and may form relatively extended energy-deposition tracks [31,32]. Photons, by contrast, deposit energy indirectly through secondary charged particles generated in processes such as the photoelectric effect, Compton scattering, and, at sufficiently high energies, pair production [31,33]. Neutrons follow a different interaction pathway and can give rise to recoil events or secondary particles that redistribute energy in a manner unlike that observed for charged particles or photons [31,34,35]. As a result, even when the target material remains the same, the microscopic structure of radiation-induced events may vary substantially depending on the type of incident particle.

For this reason, a meaningful description of radiation interaction in MAPbI_3_ should go beyond integral or purely qualitative characteristics and include a spatially resolved analysis of deposited energy together with the identification of the underlying interaction channels. Such an approach makes it possible to relate particle type and irradiation conditions to local energy-release patterns and event morphology, thereby providing a more complete physical picture of the material response.

Monte Carlo particle-transport simulation is particularly well suited for this purpose, since it allows one to follow the full cascade of primary and secondary interactions in a realistic material model. In this context, Geant4 [36,37] offers a convenient and flexible framework for defining the target geometry, material composition, source parameters, and relevant physics processes, while also enabling detailed event-level scoring and track analysis. This makes it possible to examine not only the overall deposited energy, but also its spatial distribution and the role of individual physical mechanisms in shaping that distribution.

In the present work, we consider a single-layer MAPbI_3_ target as a model system and use Geant4 to investigate its interaction with incident radiation. The emphasis is placed on deposited energy as a spatially resolved quantity and on the contribution of different physical processes to the observed deposition patterns. The aim is to clarify how particle type, interaction mechanism, and local energy-deposition behavior are related in this material, thereby providing a more detailed physical basis for understanding radiation interaction in hybrid halide perovskites.

Although the general interaction mechanisms of electrons, photons, and neutrons are well established in radiation physics, their relative role in MAPbI_3_ and APbI_3_-based detector structures is not determined only by the type of projectile. It also depends on the high-Z Pb/I framework, the finite absorber thickness, the generation and transport of secondary particles, and the spatial localization of deposited energy. Previous Geant4 simulations of APbI_3_ perovskites under space relevant irradiation showed that secondary particle generation can differ noticeably between MAPbI_3_, FAPbI_3_, CsPbI_3_, and mixed cation compositions, which makes the separation of primary and secondary contributions important for interpreting radiation response [38]. The novelty of the present work is therefore not the identification of electron, photon, or neutron interaction mechanisms in general, but their unified step-resolved comparison in the same MAPbI_3_ material model and in simplified MAPbI_3_-based detector stacks. This makes it possible to distinguish direct charged-particle stopping, photon-induced secondary-electron cascades, and neutron-induced localized secondary-particle hotspots under identical scoring and visualization conditions.

## 2. Calculation Details

### 2.1. Geometry and Irradiation Conditions

The simulations were performed for a MAPbI_3_ target represented, in the baseline case, by a single perovskite layer with a thickness of 1 mm. This geometry does not correspond to the actual thickness of active perovskite layers in most photovoltaic or detector devices, which are typically much thinner. However, the use of a thicker model target in the present work was intentional. Since the main purpose of the study was to analyze the spatial distribution of deposited energy, event structure, and the contributions of individual interaction processes in the perovskite itself, the thick-layer approximation provides substantially better event statistics within a reasonable computational time. In this way, the number of interaction events inside the modeled material can be increased without introducing extremely large primary fluences, which would make the calculations unnecessarily expensive. The adopted approach is therefore intended to provide a clearer material-level picture of radiation interaction in MAPbI_3_, rather than a direct one-to-one reproduction of a specific thin-film device.

Consequently, the absolute interaction probabilities, deposited-energy values, and hotspot intensities obtained for the 1 mm absorber should not be directly transferred to submicron perovskite solar-cell layers. In thinner films, the escape probability of primary and secondary particles, the fraction of energy deposited inside the active layer, and the role of interfaces may differ substantially. Therefore, the single-layer model is used here mainly to identify material-level transport regimes and spatial energy-deposition morphologies, while device-level degradation or lifetime predictions would require geometry-specific simulations.

In addition to the single-layer configuration, a multilayer perovskite-based radiation-detector geometry was also considered in supplementary calculations. This extended model was introduced to examine how additional structural layers may modify the transport of primary and secondary particles and alter the corresponding energy-deposition pattern. Nevertheless, the main emphasis of the present work remains on the single-layer MAPbI_3_ target, which was used as the reference system for the comparative analysis.

Three classes of incident radiation were considered: electrons, gamma photons, and neutrons. For gamma irradiation, monoenergetic sources with energies of 100 keV, 186.2 keV, 356 keV, 662 keV, 1.17 MeV, and 1.33 MeV were used. These values were chosen to represent both low-energy X-ray/gamma conditions and commonly used reference gamma-emitting radionuclides. For electrons, the selected energies were 100 keV, 600 keV, 1 MeV, 5 MeV, 8.2 MeV, and 10 MeV. The values of 0.6, 1, and 5 MeV are consistent with the range commonly used in space-related solar-cell qualification studies, where electron irradiation protocols are defined in the 0.5–5 MeV interval and suggested values are given for 0.6, 1.0, and 5.0 MeV [39]. The higher energies of 8.2 and 10 MeV were additionally included to reflect the characteristics of a practically available linear accelerator [40]. For neutrons, the energies 0.1, 0.5, 1, 2, 5, and 14 MeV were selected as representative points spanning the fast-neutron range and enabling comparison between lower- and higher-energy neutron interactions.

For the application-oriented multilayer simulations, detector inspired MAPbI_3_ containing geometries were selected as representative APbI_3_-based structures to evaluate how additional layers modify particle transport and energy deposition compared with the single-layer MAPbI_3_ model. For photon irradiation, a MAPbI_3_ photovoltaic-mode X-ray detector architecture was used as the reference case [41]. This geometry follows recent MAPbI_3_ single-crystal X-ray imaging detectors, in which thick MAPbI_3_ films up to 300 µm were grown on ITO/PTAA substrates and completed with C60/BCP/Cu contacts. In the present model, the stack was defined as ITO/PTAA/MAPbI_3_/C60/BCP/Cu, with layer thicknesses of 100 nm, 30 nm, 300 µm, 20 nm, 3 nm, and 80 nm, respectively. The MAPbI_3_ layer served as the active radiation-sensitive absorber, while the surrounding layers represented the charge-selective contacts and electrodes of the detector architecture.

The same MAPbI_3_-based X-ray/gamma detector stack was also used for electron irradiation. This choice was made to evaluate the electron-induced response of the same multilayer detector geometry, rather than introducing a separate thin-film betavoltaic structure with a much smaller active volume. Although the ITO/PTAA/MAPbI_3_/C60/BCP/Cu architecture is primarily motivated by photon detection, incident electrons can also deposit energy directly in the active MAPbI_3_ layer and in the adjacent functional layers. Therefore, this case should be interpreted as the response of a realistic APbI_3_-based detector stack under electron irradiation, rather than as an optimized electron detector.

For neutron irradiation, a different multilayer geometry was used. It was based on a reported fast-neutron perovskite detector [42] employing methylhydrazinium lead chloride (MHyPbCl_3_), as the active material. The reference detector stack consisted of Cu/BCP/C/MHyPbCl_3_/Au, where the hydrogen-containing perovskite was used to convert fast-neutron energy into recoil-proton energy through elastic neutron–hydrogen scattering. In the present work, this architecture was adapted to the APbI_3_ material system by replacing the MHyPbCl_3_ active layer with MAPbI_3_ while preserving the full multilayer sequence. The neutron-case geometry was therefore defined as Cu/BCP/C/MAPbI_3_/Au, with thicknesses of 80 nm, 6 nm, 30 nm, 0.7 mm, and 80 nm, respectively. This model should be understood as an MAPbI_3_-adapted neutron-response geometry rather than a fully optimized neutron detector.

The multilayer detector architectures are presented in the Supplementary Information (SI, Figure S1).

For the representative multilayer calculations, the selected incident energies were 662 keV photons, 1 MeV electrons, and 2 MeV neutrons. The 662 keV photon energy corresponds to the ^137^Cs gamma line and provides a standard reference case for gamma-response studies. The 1 MeV electron energy was chosen as a representative charged-particle case that can penetrate the entrance contact layers while still producing appreciable energy deposition in the MAPbI_3_ absorber. The 2 MeV neutron energy was selected as a representative fast-neutron case, allowing analysis of neutron-induced secondary-particle generation and energy deposition in the adapted multilayer structure.

For each irradiation condition, 10,000 primary particles were simulated. The same number of primary particles was used for all irradiation conditions. The objective of the present work is comparative analysis of spatial interaction morphology and dominant transport mechanisms rather than the determination of statistically exact hotspot intensities. Therefore, the maps are interpreted primarily in terms of stable spatial features and relative trends between irradiation conditions. The source was modeled as point-like to keep the irradiation geometry simple and reproducible and to avoid additional broadening associated with an extended beam spot. This choice makes the interpretation of the results more straightforward, since the observed differences in deposition patterns can be attributed primarily to particle type, particle energy, and the underlying interaction physics rather than to source-shape effects.

The particles were emitted from a point-like source located on the beam axis at the entrance side of the target and propagated normally to the layer along the X direction. The 1 mm MAPbI_3_ target was treated as a 1 × 1 × 1 mm^3^ scoring volume, and the maps shown below correspond to projections of the recorded steps onto the X-Y plane. The same number of primary particles was used for all irradiation conditions, so differences between the maps reflect changes in particle transport and interaction physics rather than differences in the simulated fluence.

### 2.2. Physics Models and Simulation Settings

The simulations were carried out in the Geant4 (version 11.3.2) framework. The material definitions included elemental composition (CH_3_NH_3_PbI_3_), mass fractions, density of 4.15 g/cm^3^, and geometric dimensions of the modeled layers. Electromagnetic interactions were treated using a standard high-accuracy physics configuration suitable for charged particles and photons over a broad energy range [43,44,45]. In particular, the electromagnetic setup included Rayleigh scattering, Compton scattering, the photoelectric effect, gamma conversion, ionization, bremsstrahlung, multiple scattering [43,46,47], and related transport processes [45]. In the available implementation, multiple scattering for electrons and positrons was handled using the Wentzel VI approach [43,44,45,48], while neutron transport was supplemented by high-precision elastic and inelastic hadronic models [44,49,50]. Radioactive decay [51], particle decay, charged hadron stopping [52], and ion transport options were also enabled in the extended physics configuration. In addition, atomic relaxation effects, including fluorescence and Auger cascades [53], were taken into account. These settings were chosen to preserve a sufficiently detailed description of both primary and secondary radiation transport in the energy range relevant to the present study.

The interaction probabilities in Geant4 are determined by the selected physics models and by the evaluated data libraries (e.g., EPDL97, G4NDL, G4PARTICLEXS, ENDF/B) used by these models. In particular, low-energy electromagnetic models use evaluated photon, electron, and atomic data, while high-precision neutron transport uses evaluated neutron data for cross sections, angular distributions, and final states. Therefore, the recorded interaction probabilities in the present work are based on the standard evaluated data implemented in Geant4. At the same time, the event numbers reported here should not be interpreted as tabulated microscopic cross sections or as a direct NIST or END database benchmark, since they also depend on target thickness, source geometry, production cuts, and the scoring procedure.

The production thresholds were defined through Geant4 range cuts and therefore depended on the material and geometry. Two production cut settings were used in the single-layer MAPbI_3_ calculations. A production range cut of 0.5 mm was used for low-energy transport cases. In MAPbI_3_, this corresponds approximately to production thresholds of 30 keV for photons, 396 keV for electrons, 380 keV for positrons, and 50 keV for protons. A production range cut of 1 mm was used for the photon and neutron series and for higher energy transport cases. In MAPbI_3_, this corresponds approximately to production thresholds of 47 keV, 659 keV, 627 keV, and 100 keV for photons, electrons, positrons, and protons, respectively. In the multilayer geometries, the range cuts and scoring discretization were reduced according to the characteristic thicknesses of the corresponding layers to avoid using a production scale larger than the modeled layer thickness.

These values define the threshold for explicit secondary particle production and should not be interpreted as tracking cutoffs. Particles below the corresponding production thresholds were not generated as separate secondary tracks, while their energy contribution was deposited locally. Therefore, the present simulations should be regarded as transport-level calculations of deposited energy distributions and explicitly generated secondary particles. They are not intended to resolve the real nanometer-scale structure of low-energy electron cascades, calculate nanodosimetric quantities, or directly predict defect formation, ion migration, phase instability, or device degradation.

The lower and upper energy bounds of the transport calculations were set to 10 eV and 100 MeV, respectively. This interval was sufficient to describe the primary radiation considered here together with the secondary particles generated inside the material. Since the main goal of the work was to analyze deposited energy and interaction channels rather than to derive defect populations directly, the simulations were focused on detailed event transport and energy-release patterns in the target volume.

### 2.3. Data Recording and Post-Processing

The Geant4 output was written in a step-resolved form. In the process-resolved analysis, the process name corresponds to the Geant4 process assigned to the recorded step. Therefore, energy listed under multiple scattering should not be interpreted as energy loss caused by multiple scattering itself. For charged particles, it means that ionization energy loss was accumulated during a step that was classified or limited by the multiple scattering transport process. This distinction is important when interpreting process-resolved tables and prevents overestimating multiple scattering as an independent energy deposition channel. For each recorded step, the particle identity, track and parent identifiers, event number, spatial coordinates, kinetic energy, local energy loss, step length, track length, interaction process, and traversed volume were stored in the output log and then processed in Python (version 3.13.3). This made it possible to analyze both event-wise and process-resolved energy deposition behavior.

For the visualization of spatial energy-release patterns, adaptive density field maps were constructed in Python. In the implemented workflow, track segments were first reconstructed from successive Geant4 steps and, when necessary, interpolated along the trajectory with a characteristic subdivision length of 0.1 mm in order to distribute the signal more continuously in space. The adaptive field itself was then estimated from local neighborhoods using a k-nearest-neighbor [54] procedure, with the local signal scaled by the area associated with the distance to the outer neighbor.

For event density maps, the plotted quantity is a normalized dimensionless density field. Each panel was normalized independently to the interval from 0 to 1 after logarithmic scaling, so these maps should be used to compare spatial morphology rather than absolute event numbers. Absolute event statistics are presented separately in the integral plots and Supplementary Tables S1 and S2.

For deposited energy maps, the local signal was calculated from the sum of Geant4 step energy losses projected onto the X-Y plane and divided by the corresponding projected area. Unless otherwise stated, the color scale represents the total deposited energy density accumulated for 10,000 primary particles, in MeV mm^−2^. The values are therefore normalized by projected area but not by the number of primary particles or by the number of recorded events. This convention was used to preserve the absolute scale of local energy release hotspots while keeping the same primary particle statistics for all irradiation conditions.

To improve the stability of sparse projections, the mapping routine also used support masks and Gaussian smoothing, and when the original weighted field contained too little signal, a fallback “phantom” representation based on the underlying count distribution was generated for visualization. The final rendering parameters for the maps were selected automatically from the distribution of positive field values using quantile-based limits, with power-law normalization applied when the dynamic range became too large. This made it possible to compare projections with strongly different local intensities without losing low-signal regions.

The post processing procedure was used only for visualization of the Geant4 step output. Its purpose was to convert separate step points and local clusters into continuous and readable spatial regions. Without this procedure, the maps would appear as sparse point distributions, which would make comparison of energy release morphology less clear. Interpolation and smoothing do not change the total deposited energy, event numbers, or process-resolved statistics obtained from the original Geant4 output. However, they can affect the apparent size, contrast, and sharpness of individual hotspots in the rendered figures. This effect is most important for photon and neutron irradiation, where rare localized events contribute strongly to the visual pattern. Therefore, hotspots in the maps should be interpreted as qualitative indicators of localized energy release and spatial morphology. Their apparent diameter, exact shape, and peak color value should not be interpreted as direct physical hotspot sizes or nanodosimetric peak doses.

Cluster-like spatial structures were assessed automatically. In the Python analysis code, clustering was attempted only when the point distribution was sufficiently inhomogeneous according to a k-nearest-neighbor dispersion criterion. If this condition was satisfied, HDBSCAN [55] was used when available; otherwise, a DBSCAN-based [56] fallback was applied, with the neighborhood radius estimated from the empirical distribution of nearest-neighbor distances.

Several normalization modes were used in the statistical analysis of spectra and event distributions, including raw counts, normalization per particle, normalization by the total number of steps or events, and probability-density normalization. This allowed the same dataset to be viewed either in absolute terms or in forms more suitable for comparison between different irradiation conditions.

## 3. Results and Discussion

Below we discuss the spatial distributions of events associated with primary particles, the distributions of secondary-particle events, the corresponding maps of deposited energy, and the contributions of individual physical processes. Taken together, these characteristics provide a more complete picture of how different types of incident radiation interact with the perovskite target. Such analysis is relevant not only for the general understanding of radiation–matter interaction in hybrid halide perovskites, but also for the interpretation of possible device-level implications. In practical structures, localized energy deposition may affect the active region in different ways depending on the application. In detector-oriented systems, it is directly related to signal generation, whereas in other device configurations it may indicate regions where radiation-related excitation and local energy release are concentrated. For this reason, the comparison presented below is aimed not at directly predicting defect formation or device degradation, but at clarifying how particle type affects the spatial morphology of interaction events and deposited energy patterns.

### 3.1. Electron Irradiation

Under electron irradiation, the interaction pattern in MAPbI_3_ is governed by continuous energy loss, multiple Coulomb scattering, and the generation of secondary electrons. In contrast to photon irradiation, where primary quanta may travel relatively long distances before interacting, incident electrons begin to lose energy and change direction from the earliest stages of transport. As a result, the spatial structure of interaction events is strongly energy-dependent and evolves from a compact near-surface region to an extended forward-directed pattern as the electron energy increases.

**Figure 1:**
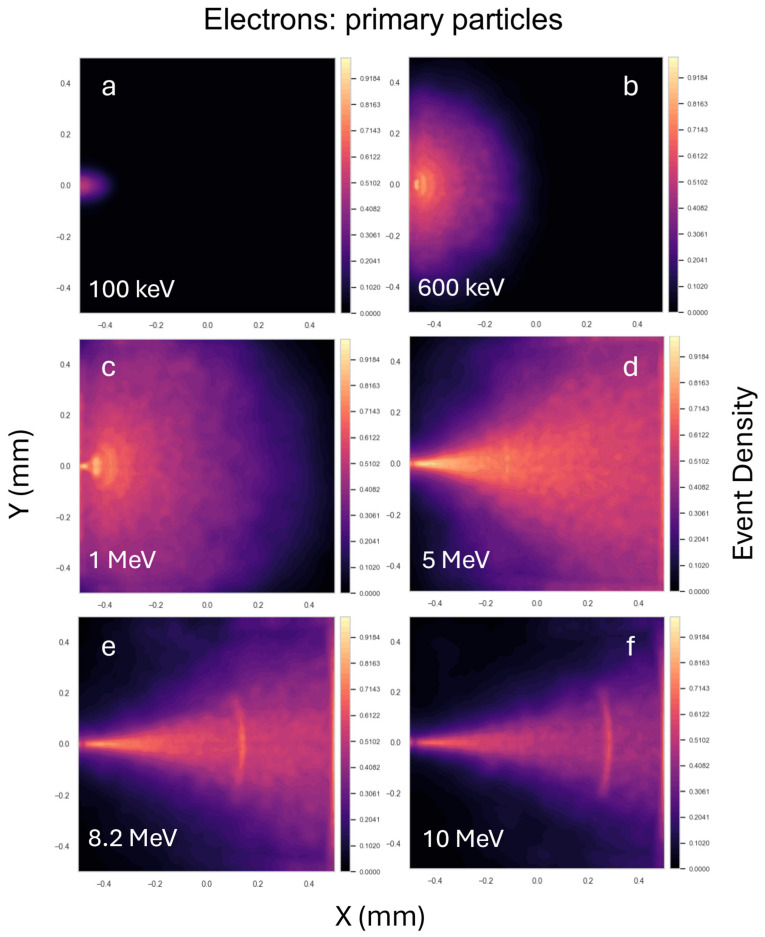
Heat maps of interaction events associated with primary electrons in a 1 mm thick MAPbI_3_ layer for incident-electron energies of (**a**) 100 keV, (**b**) 600 keV, (**c**) 1 MeV, (**d**) 5 MeV, (**e**) 8.2 MeV, and (**f**) 10 MeV.

Figure 1 shows the heat maps of interaction events associated with primary electrons in the 1 mm thick MAPbI_3_ layer. At 100 keV (Figure 1a), the events are confined to a small region near the entrance surface, which is consistent with the short penetration depth of low-energy electrons and their rapid loss of directionality due to strong multiple scattering.

At 600 keV (Figure 1b), the interaction region becomes substantially broader and extends deeper into the layer, although the highest event density is still concentrated in the front part of the target. At 1 MeV (Figure 1c), the distribution occupies a significant fraction of the material volume, indicating a clear increase in penetration depth and a more spatially developed transport regime.

A further increase in electron energy leads to the formation of an elongated forward-directed interaction structure. At 5 MeV (Figure 1d), the event pattern becomes distinctly stretched along the incident-particle direction, reflecting the larger electron range and the reduced importance of early stopping near the entrance surface. At 8.2 and 10 MeV (Figure 1e,f), the interaction region extends through almost the full thickness of the absorber and acquires the form of a narrow conical or beam-like domain. In these cases, a substantial fraction of electrons preserves its forward direction over longer distances, while local regions of enhanced event density appear deeper in the material, indicating cumulative scattering and intensified energy-loss processes along the track. Overall, the primary-electron maps demonstrate a clear transition from a near-surface interaction regime at low energies to a through-going transport regime at multi-MeV energies.

**Figure 2:**
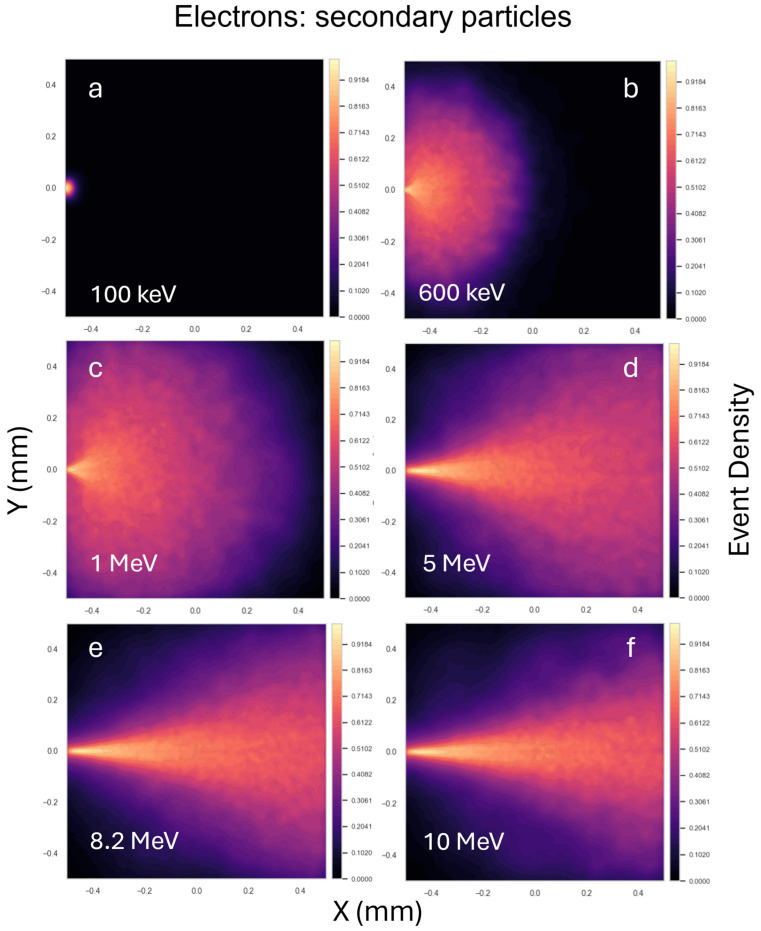
Heat maps of interaction events associated with secondary particles generated in a 1 mm thick MAPbI_3_ layer under electron irradiation with incident-electron energies of (**a**) 100 keV, (**b**) 600 keV, (**c**) 1 MeV, (**d**) 5 MeV, (**e**) 8.2 MeV, and (**f**) 10 MeV.

The corresponding spatial distributions of events associated with secondary particles generated under electron irradiation are shown in Figure 2. Even at 100 keV (Figure 2a), secondary-particle events are already observed outside the compact primary-interaction region, although their spatial extent remains limited. At 600 keV (Figure 2b), the distribution becomes much broader both laterally and in depth, indicating that the produced secondary electrons are able to transport energy over a significantly larger volume of the target. At 1 MeV (Figure 2c), the secondary-particle events occupy a considerable part of the MAPbI_3_ layer and form a well-developed diffuse interaction region.

At higher primary-electron energies, the secondary-particle distributions acquire a pronounced forward-directed morphology. At 5 MeV (Figure 2d), the events form a broad cone extending from the entrance surface toward the rear part of the absorber. At 8.2 and 10 MeV (Figure 2e,f), this cone spans nearly the entire layer thickness and becomes more spatially uniform along the beam direction, while still preserving a noticeable lateral spread.

This behavior indicates that, as the energy of the primary electrons increases, the generated secondary particles redistribute the deposited energy over an increasingly large volume of the material. Thus, the secondary-particle maps complement the primary-electron results by showing that the overall radiation response of MAPbI_3_ is determined not only by the direct transport of incident electrons, but also by the spatially extended cascade of secondary interactions they initiate.

**Figure 3:**
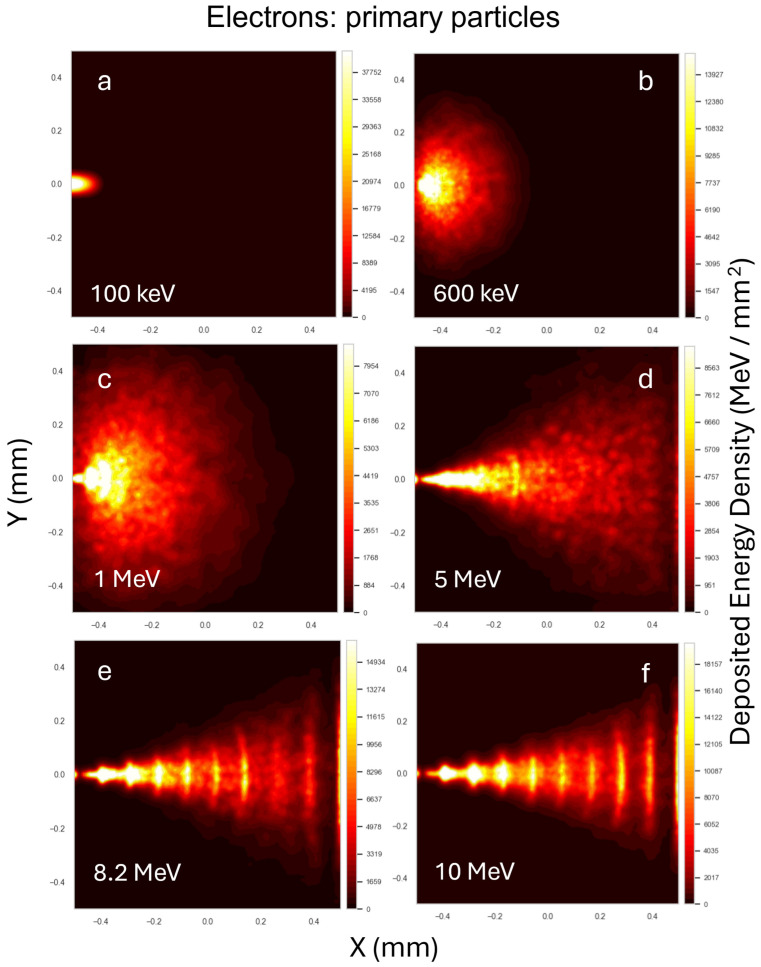
Heat maps of released energy associated with primary electrons in a 1 mm thick MAPbI_3_ layer for incident-electron energies of (**a**) 100 keV, (**b**) 600 keV, (**c**) 1 MeV, (**d**) 5 MeV, (**e**) 8.2 MeV, and (**f**) 10 MeV. The maximum values of the color scale are approximately 3.78 × 10^4^, 1.39 × 10^4^, 7.95 × 10^3^, 8.56 × 10^3^, 1.49 × 10^4^, and 1.82 × 10^4^ MeV/mm^2^, respectively.

The transport features discussed above are directly reflected in the spatial distribution of energy deposited by primary electrons (Figure 3). At low incident energies, the deposited energy is concentrated near the entrance surface, where electrons undergo rapid deceleration and lose a substantial fraction of their energy over a short distance. This behavior is most pronounced at 100 keV (Figure 3a), where the deposition pattern is confined to a compact near-surface region. At 600 keV (Figure 3b), the deposition zone becomes broader and extends deeper into the material, although the front part of the absorber still contains the highest energy-density region. At 1 MeV (Figure 3c), the deposited-energy pattern expands further into the target volume and becomes more heterogeneous, reflecting the increasing role of repeated scattering and cascade development.

A more pronounced forward-directed morphology is observed at 5 MeV (Figure 3d), where the energy deposition forms an elongated cone along the incident-electron direction. In this regime, the response is no longer dominated by near-surface stopping but reflects sustained energy transfer over a substantial fraction of the absorber thickness. At 8.2 and 10 MeV (Figure 3e,f), the deposited energy pattern extends through nearly the entire 1 mm layer and becomes more collimated along the beam axis. The maps also show local axial nonuniformities and enhanced deposition near the rear part of the absorber. These fine-scale maxima should be interpreted with caution, since at multi-MeV energies part of the visible structure may arise from the discrete treatment of electron transport, multiple scattering, and ionization processes in Geant4 rather than from a fully continuous spatial distribution of energy loss [43,44,46]. Therefore, the most robust physical trend is the overall transition from a compact entrance-localized deposition pattern at low energies to a through-going, forward-directed energy-deposition structure with increasing electron energy.

In addition to spatial morphology, the absolute values of the deposited energy density scale should be considered. The maximum values obtained from the maps are approximately 3.78 × 10^4^, 1.39 × 10^4^, 7.95 × 10^3^, 8.56 × 10^3^, 1.49 × 10^4^, and 1.82 × 10^4^ MeV/mm^2^ for incident electron energies of 100 keV, 600 keV, 1 MeV, 5 MeV, 8.2 MeV, and 10 MeV, respectively. The highest local value is therefore observed at 100 keV, where the electron energy is released within a compact near-surface region. As the energy increases to 600 keV and 1 MeV, the maximum decreases because the deposited energy is spread over a larger absorber volume. At higher energies, the local maxima increase again, mainly due to axial concentration of energy release along the electron trajectory and deeper deposition near the rear side of the layer. Overall, the most robust trend is the transition from compact entrance-localized deposition at low energies to a thoroughgoing, forward-directed energy release structure at multi-MeV energies.

**Figure 4:**
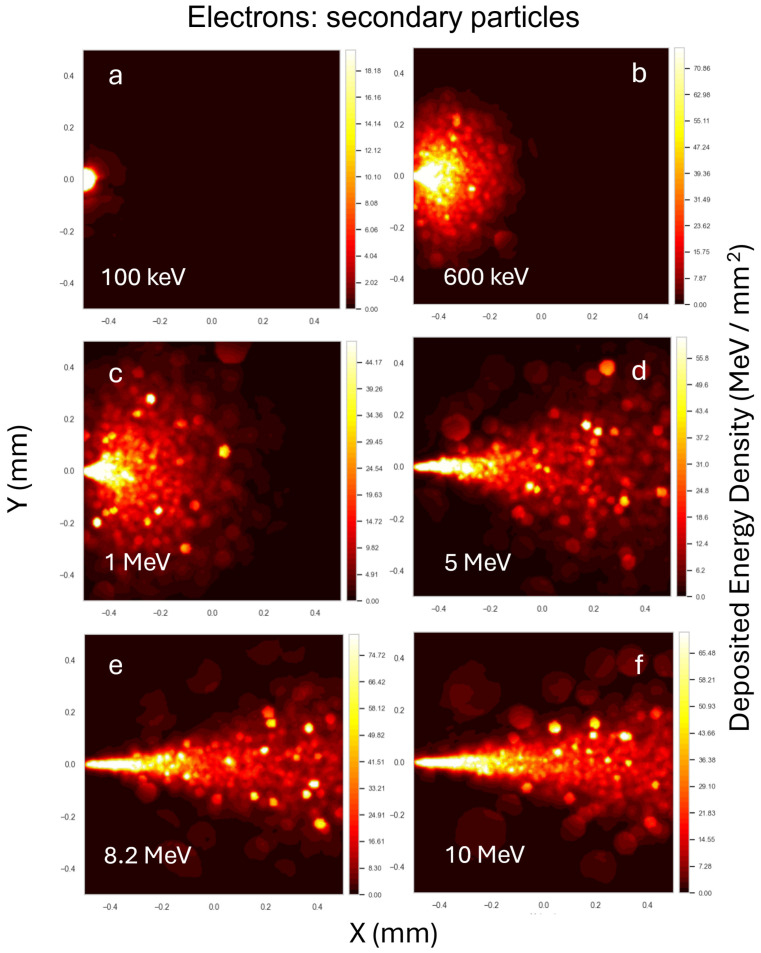
Heat maps of released energy associated with secondary particles generated in a 1 mm thick MAPbI_3_ layer under electron irradiation with incident-electron energies of (**a**) 100 keV, (**b**) 600 keV, (**c**) 1 MeV, (**d**) 5 MeV, (**e**) 8.2 MeV, and (**f**) 10 MeV. The maximum values of the color scale are approximately 18.18, 70.86, 44.17, 55.8, 74.72, and 65.48 MeV/mm^2^, respectively.

The corresponding maps of energy deposited by secondary particles generated under electron irradiation are shown in Figure 4.

Compared with the primary electron contribution, the secondary particle pattern is more heterogeneous and contains numerous localized regions of enhanced energy release. At 100 keV (Figure 4a), the energy deposition remains confined close to the entrance region, while at 600 keV and 1 MeV (Figure 4b,c) it expands both laterally and in depth, indicating more effective redistribution of energy by secondary particles.

At higher incident energies, the secondary contribution becomes increasingly extended along the incident electron direction. At 5 MeV (Figure 4d), the map shows a broad forward spreading region with strong local fluctuations. At 8.2 and 10 MeV (Figure 4e,f), the deposited energy reaches almost the full absorber thickness and remains spatially diffuse, even though the primary electron transport becomes more collimated. This indicates that secondary particles continue to redistribute energy away from the primary track and form a wider affected volume. Representative 3D trajectories illustrating this behavior are shown in Figure S2.

The absolute scale of the maps confirms the hotspot character of the secondary contribution. The maximum deposited energy density values are approximately 18.18, 70.86, 44.17, 55.8, 74.72, and 65.48 MeV/mm^2^ for 100 keV, 600 keV, 1 MeV, 5 MeV, 8.2 MeV, and 10 MeV, respectively. The lowest maximum is observed at 100 keV, where the secondary cascade remains weak and localized near the entrance region. The maximum increases sharply at 600 keV, and then remains at the same order of magnitude for higher energies, reflecting the formation of localized secondary particle hotspots within an increasingly extended deposition volume.

**Figure 5:**
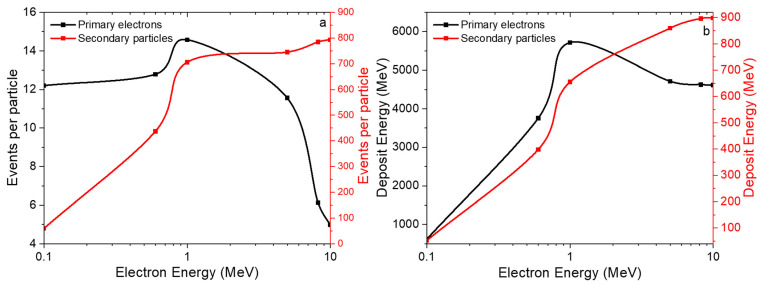
Integral characteristics of electron-perovskite interaction: (**a**) events per particle, (**b**) deposited energy.

The spatially resolved maps presented above are complemented by integral characteristics of interaction, shown in Figure 5 for both primary and secondary particles. For primary electrons, both the number of interaction events (Figure 5a) and the deposited energy (Figure 5b) increase as the incident energy rises from 100 keV to the MeV range. This behavior is associated with the increase in electron penetration depth. Low-energy electrons are stopped close to the entrance surface, so only a limited part of the absorber is involved in energy transfer. As the energy increases, electrons travel through a larger fraction of the 1 mm MAPbI_3_ layer, producing more interaction steps and depositing energy over a larger volume.

The maximum in the MeV range indicates an optimal balance between penetration and stopping inside the absorber. In this regime, electrons are energetic enough to reach deep regions of the layer, but they still undergo efficient scattering and ionization within the material. At higher energies, this balance changes. A larger fraction of primary electrons preserves its forward direction and can pass through the layer without releasing all of its energy inside the simulated volume. As a result, the number of recorded interaction events and the deposited energy no longer increase proportionally with incident energy and tend toward saturation or a slight decrease.

This behavior is important for interpreting the energy maps. The local maxima observed at 8.2 and 10 MeV do not necessarily mean that the total stopping efficiency of the layer continues to grow. Instead, they indicate that energy release becomes more concentrated along the beam direction and in deeper regions of the absorber, while part of the incident electron energy may be carried beyond the 1 mm layer. Thus, the integral curves and spatial maps describe complementary aspects of the same transition: from compact stopping at low energies to deeper, more directional transport at higher energies.

In contrast, the contribution of secondary particles shows a more gradual increase with energy for both event numbers (Figure 5a) and deposited energy (Figure 5b). At higher energies, this contribution approaches saturation, indicating that the secondary cascade becomes more developed but remains limited by the finite thickness of the absorber. The energy deposited by secondary particles is noticeably lower than that deposited by primary electrons and accounts for about 10% of the total deposited energy. Therefore, secondary particles do not dominate the energy balance under electron irradiation, but they are important for broadening the affected volume and increasing the spatial heterogeneity of energy deposition.

Taken together with the spatial maps, these results demonstrate that the overall interaction behavior in MAPbI_3_ under electron irradiation is governed by the interplay between direct interactions of primary electrons and cascade-mediated processes involving secondary particles. While primary electrons determine the general penetration depth and directionality of transport, secondary particles play a key role in increasing the number of interaction events and spreading the released energy over a larger volume.

The Supplementary Information contains process-resolved integral statistics used to support the identification of the dominant interaction mechanisms (Figures S3 and S4). Detailed spatial maps for individual processes are not discussed here, since the main article focuses on the spatial morphology of total primary and secondary particle contributions.

### 3.2. Photon Irradiation

In contrast to electron irradiation, where the spatial structure of the interaction is governed by continuous energy loss and multiple scattering of charged particles, photon irradiation is characterized by a fundamentally different transport mechanism. Due to their neutral nature, γ-quanta interact with matter relatively weakly and may propagate over significant distances without interaction. As a result, the number of interaction events associated with primary photons remains limited, and the overall response of the material is largely determined by the generation of secondary charged particles.

**Figure 6:**
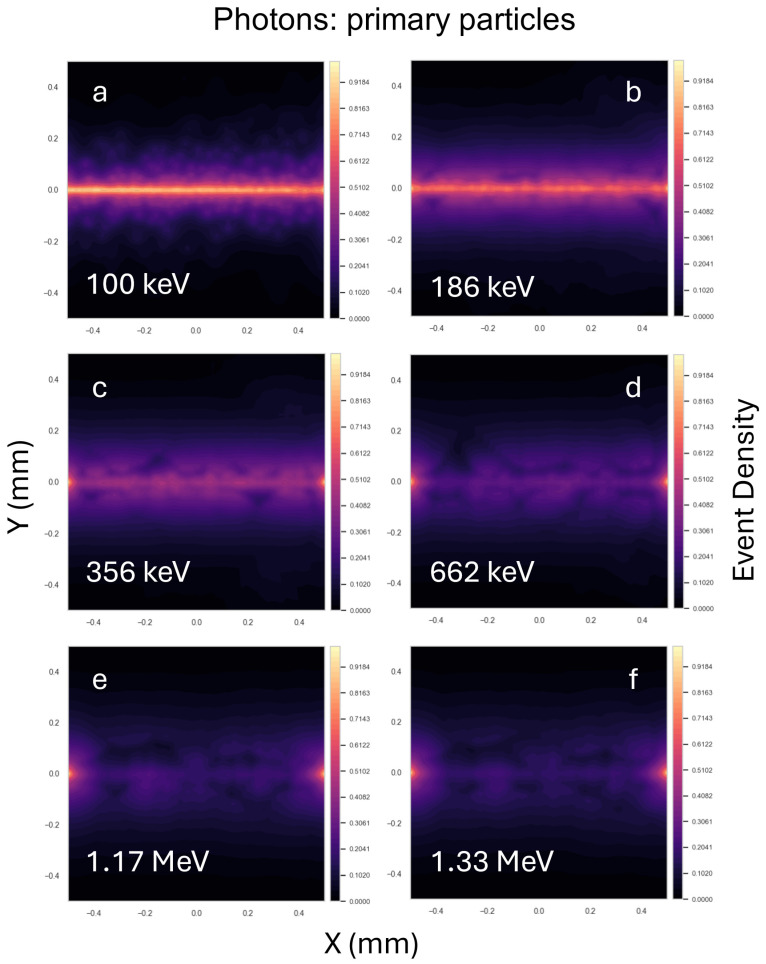
Heat maps of interaction events associated with primary photons in a 1 mm thick MAPbI_3_ layer for incident energies of (**a**) 100 keV, (**b**) 186 keV, (**c**) 356 keV, (**d**) 662 keV, (**e**) 1.17 MeV, and (**f**) 1.33 MeV.

This behavior is reflected in the spatial distributions of interaction events for primary photons (Figure 6). The events are confined to a narrow region connecting the entrance and exit surfaces of the 1 mm thick MAPbI_3_ layer, indicating that most photons traverse the material with only a few interactions. With increasing photon energy from 100 keV to the MeV range, the probability of interaction per unit path length decreases, and the already sparse distribution of primary events becomes even more localized along the beam direction.

**Figure 7:**
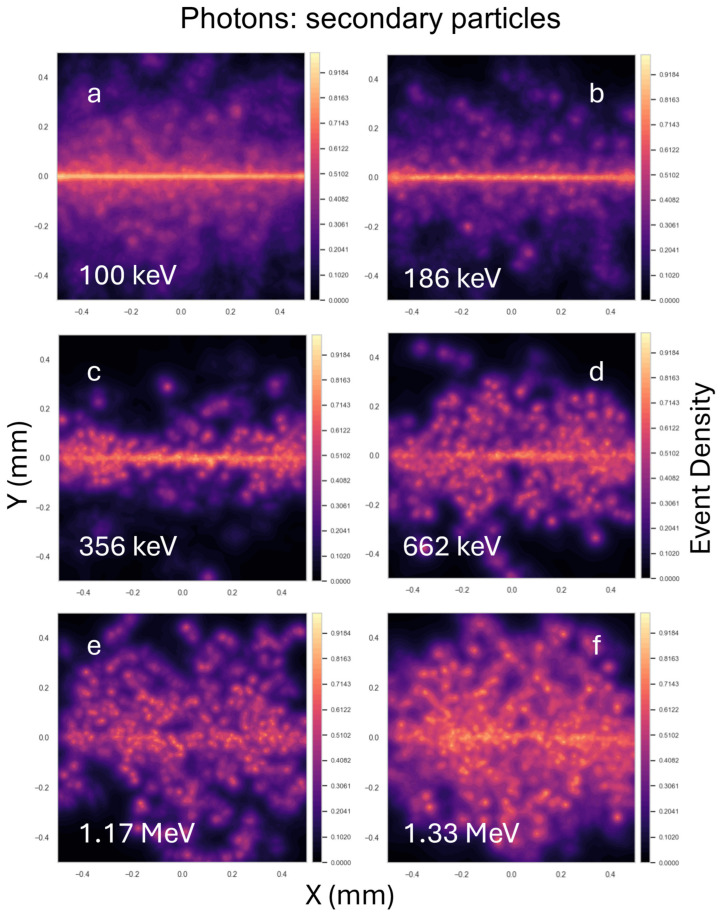
Heat maps of interaction events associated with secondary particles in a 1 mm thick MAPbI_3_ layer for incident energies of (**a**) 100 keV, (**b**) 186 keV, (**c**) 356 keV, (**d**) 662 keV, (**e**) 1.17 MeV, and (**f**) 1.33 MeV.

The corresponding spatial distributions of interaction events for secondary particles are shown in Figure 7. In contrast to the primary photons, secondary particles form a significantly more extended interaction pattern throughout the material.

In the intermediate-energy range (186–662 keV), a noticeable lateral broadening of the event distribution is observed, reflecting the increasing ranges of secondary electrons and their multiple scattering. As the photon energy increases further, the spatial structure becomes more developed, indicating more efficient cascade formation.

**Figure 8:**
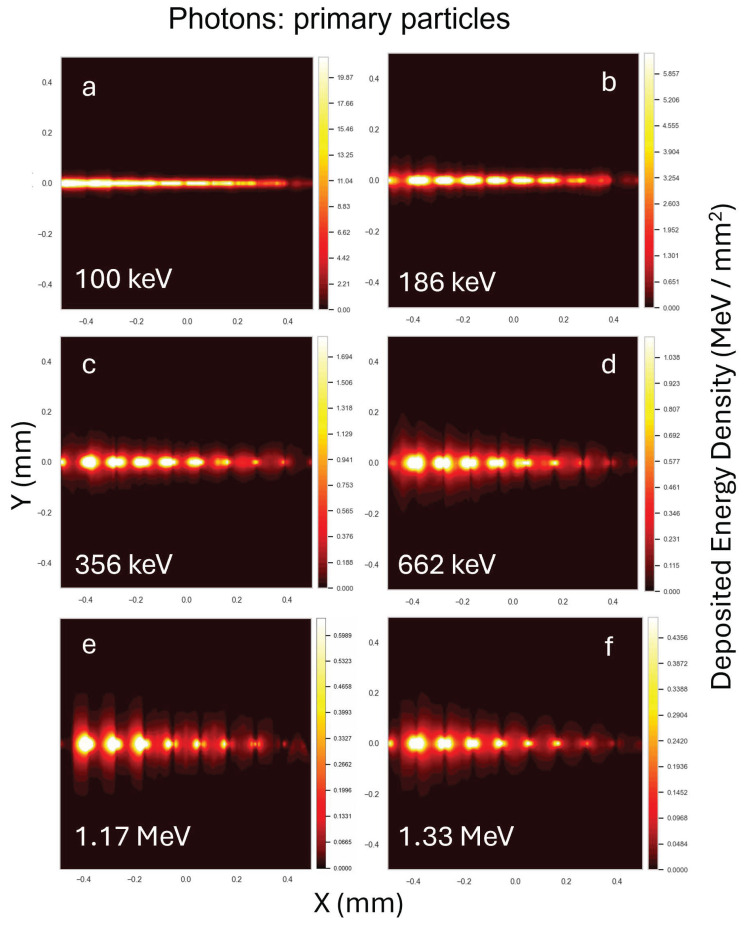
Heat maps of released energy associated with primary photons in a 1 mm thick MAPbI_3_ layer for incident energies of (**a**) 100 keV, (**b**) 186 keV, (**c**) 356 keV, (**d**) 662 keV, (**e**) 1.17 MeV, and (**f**) 1.33 MeV. The maximum color scale values are approximately 19.87, 5.86, 1.69, 1.04, 0.599, and 0.436 MeV/mm^2^, respectively.

The spatial distributions of deposited energy for primary photons are shown in Figure 8. These maps closely follow the corresponding event distributions and remain concentrated along the incident direction, since primary photons transfer energy only through discrete interactions. The maximum values of the deposited energy density scale are approximately 19.87, 5.86, 1.69, 1.04, 0.599, and 0.436 MeV/mm^2^ for photon energies of 100 keV, 186.2 keV, 356 keV, 662 keV, 1.17 MeV, and 1.33 MeV, respectively. Thus, the direct photon contribution decreases strongly with increasing energy. This reflects the lower probability of local energy transfer by primary photons in the MeV range and confirms that their direct contribution does not form a broad volumetric deposition pattern.

**Figure 9:**
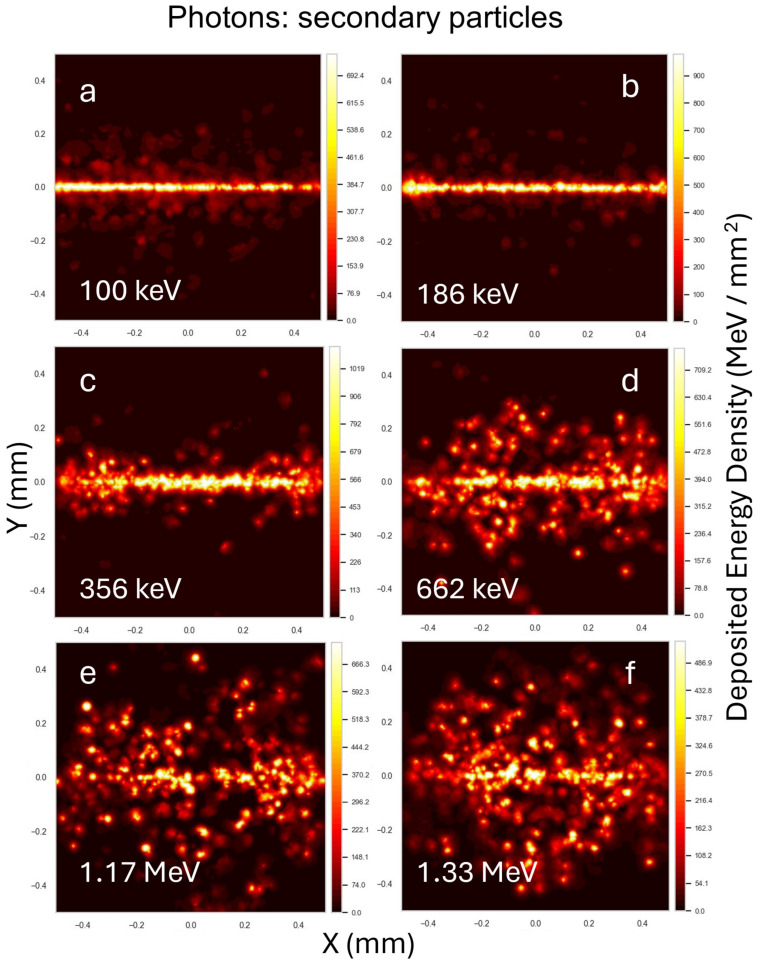
Heat maps of released energy associated with secondary particles in a 1 mm thick MAPbI_3_ layer for incident energies of (**a**) 100 keV, (**b**) 186 keV, (**c**) 356 keV, (**d**) 662 keV, (**e**) 1.17 MeV, and (**f**) 1.33 MeV. The maximum color scale values are approximately 692.4, 1000, 1019, 709.2, 666.3, and 486.9 MeV/mm^2^, respectively.

In contrast, the deposited energy maps for secondary particles (Figure 9) show much larger absolute values and a more heterogeneous spatial structure. The maximum scale values are approximately 692.4, 1000, 1019, 709.2, 666.3, and 486.9 MeV/mm^2^ for 100 keV, 186.2 keV, 356 keV, 662 keV, 1.17 MeV, and 1.33 MeV, respectively. The highest local energy release is observed in the low and intermediate photon energy range, where photoelectric absorption and Compton scattering efficiently generate secondary electrons that deposit energy over short distances. At higher photon energies, the distribution becomes more spatially extended and fragmented, while the local maximum decreases. This indicates that the energy is redistributed over a larger volume by higher energy secondary particles rather than being concentrated in a compact region. Therefore, photon irradiation is characterized by weak direct energy deposition by primary photons and a dominant secondary particle contribution to the actual energy release pattern.

**Figure 10:**
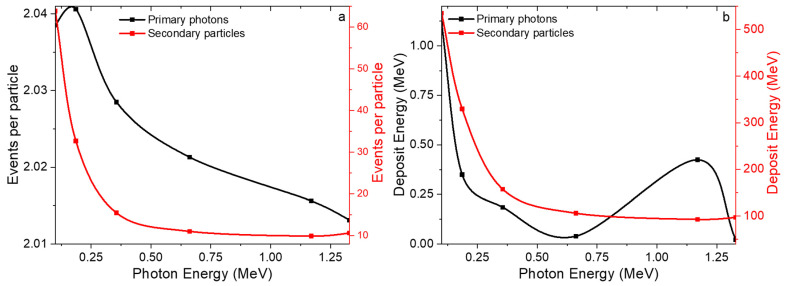
Integral characteristics of photon-perovskite interaction: (**a**) events per particle, (**b**) deposited energy.

The integral characteristics of photon interaction in MAPbI_3_ are summarized in Figure 10, showing both the average number of interaction events per particle and the corresponding released energy for primary photons and secondary particles.

For primary photons (Figure 10a,b), the number of recorded interactions remains low over the entire energy range. This indicates that most gamma quanta traverse the 1 mm MAPbI_3_ layer without frequent interactions. At the same time, the directly deposited energy decreases strongly with increasing photon energy. This trend is consistent with the lower probability of local photon absorption in the MeV range and with the transition from more efficient low-energy photon absorption to a regime where photons mainly initiate secondary particle cascades.

The secondary particle contribution (Figure 10a,b) shows a different behavior. Both the number of secondary events and the deposited energy decrease with increasing photon energy, but their absolute contribution remains much larger than that of primary photons. This means that the detector response under photon irradiation is not controlled by direct energy deposition from gamma quanta. Instead, it is governed by secondary electrons and related deexcitation products generated after rare primary photon interactions. The decrease in secondary event number at higher photon energies can be associated with longer particle ranges and lower local interaction density, so the deposited energy becomes distributed over a larger volume rather than concentrated in a small region. Representative 3D trajectories are shown in Figure S5.

Thus, the integral characteristics confirm the conclusion drawn from the spatial maps: photon irradiation produces a weak primary response, while the main energy deposition is formed by secondary particle transport. In this sense, primary photons act mainly as cascade initiators, whereas secondary charged particles determine the actual spatial and energetic response of MAPbI_3_.

Additional process-specific integral characteristics for photon irradiation are provided in Supplementary Information (Figures S6 and S7). Only integral process contributions are included there; process-resolved spatial maps are not presented, since the present work focuses on the total primary and secondary particle response.

### 3.3. Neutron Irradiation

While both electron and photon irradiation are governed primarily by electromagnetic interactions, neutron irradiation introduces a fundamentally different interaction mechanism. Due to the absence of electric charge, neutrons do not undergo continuous energy loss through ionization and instead interact with matter via nuclear processes, including elastic and inelastic scattering, as well as nuclear reactions. As a result, the spatial and energetic characteristics of neutron-induced effects differ significantly from those observed for charged particles and photons.

In contrast to electron and photon irradiation, the interaction of neutrons with matter is governed by fundamentally different physical mechanisms. Being electrically neutral, neutrons do not undergo continuous energy loss via ionization along their trajectory. Instead, their transport is defined by discrete nuclear interactions, including elastic scattering, inelastic reactions, and energy-dependent channels such as (n,γ), (n,p), (n,α), and multi-particle emission. As a result, the spatial structure of radiation response is not associated with a continuous track of the primary particle, but rather with the locations of individual interaction events and the subsequent propagation of secondary particles.

Neutron capture on iodine is, in principle, possible and can lead to the formation of an excited compound nucleus followed by emission of photon and subsequent electromagnetic energy deposition. However, the neutron energies considered in the present work correspond mainly to the fast neutron range. Under these conditions, radiative capture is not expected to dominate the neutron response, since capture is most efficient for thermal and resonance energy neutrons. In the present simulations, the main spatial features of neutron-induced energy release are therefore attributed primarily to elastic and inelastic nuclear interactions, recoil particles, and secondary charged products. A complete isotope resolved analysis of iodine capture and other individual reaction yields is beyond the scope of the present work.

**Figure 11:**
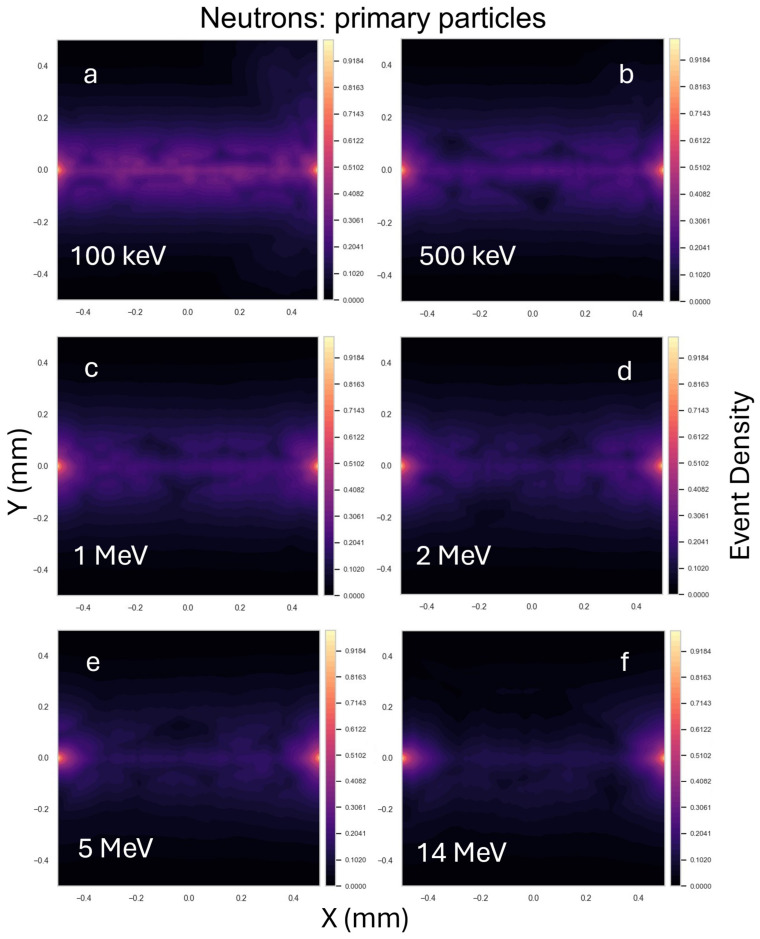
Heat maps of interaction events associated with primary neutrons in a 1 mm thick MAPbI_3_ layer for incident energies of (**a**) 0.1 MeV, (**b**) 0.5 MeV, (**c**) 1 MeV, (**d**) 2 MeV, (**e**) 5 MeV, (**f**) 14 MeV.

This leads to a qualitatively different picture compared to electrons and photons. The spatial distributions of interaction events for primary neutrons are presented in Figure 11. At all considered energies, the distributions exhibit a weakly pronounced, elongated structure along the incident direction, reflecting the fact that neutrons propagate through the material with relatively small angular deviation and interact only at discrete points along their trajectory. Unlike charged particles, no continuous track-like energy loss is observed.

At lower neutron energies (0.1–1 MeV) (Figure 11a–c), interaction events are relatively sparse and distributed along the beam direction, with moderate spatial fluctuations. These interactions are primarily associated with elastic scattering and low-energy nuclear processes, which do not lead to strong localization of events.

As the energy increases (2–5 MeV) (Figure 11d,e), the overall spatial pattern remains similar, but becomes slightly more diffuse, reflecting the increase in neutron mean free path and the reduced probability of interaction within the finite thickness of the absorber. At the same time, the role of inelastic channels increases; however, this does not lead to a higher density of primary neutron events but rather affects the characteristics of the generated secondary particles.

At 14 MeV (Figure 11f), the distribution becomes even more uniform and weakly structured. Interaction events are spread over a larger fraction of the material volume, indicating that a significant portion of neutrons traverses the layer without interaction or interacting only once. As a result, the primary-neutron contribution is characterized by a low-density, spatially extended distribution rather than localized regions of high event concentration.

**Figure 12:**
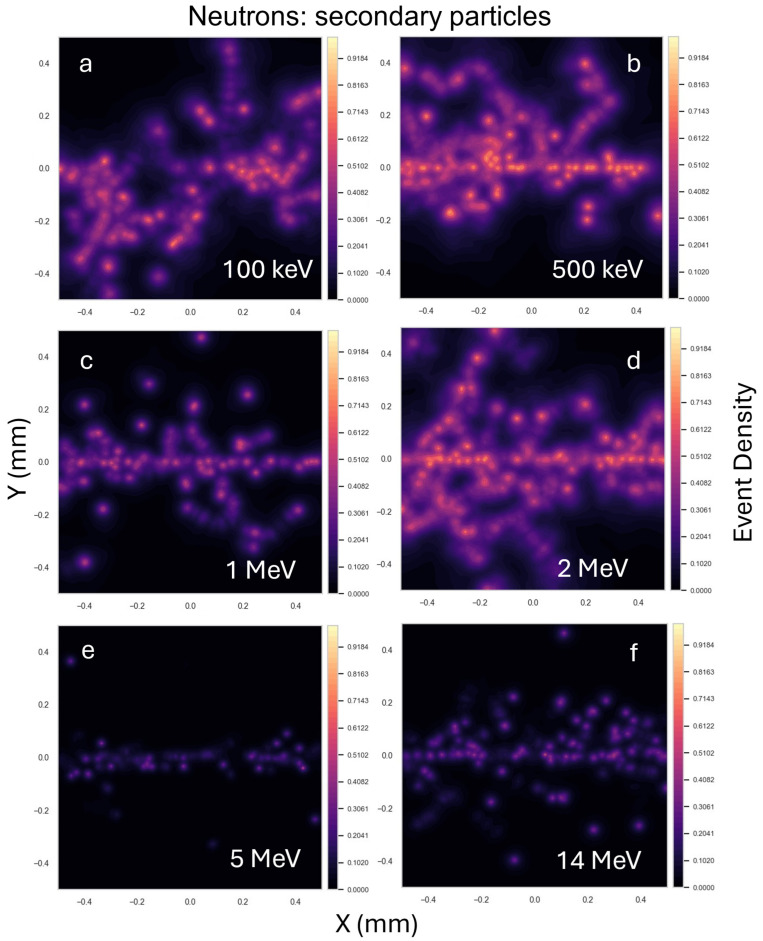
Heat maps of interaction events associated with secondary particles in a 1 mm thick MAPbI_3_ layer for incident energies of (**a**) 0.1 MeV, (**b**) 0.5 MeV, (**c**) 1 MeV, (**d**) 2 MeV, (**e**) 5 MeV, (**f**) 14 MeV.

The spatial distributions of interaction events associated with secondary particles are presented in Figure 12. In contrast to the primary-neutron maps, these distributions exhibit a significantly richer and more heterogeneous spatial structure, reflecting the fact that the dominant energy transfer under neutron irradiation is mediated by secondary charged particles.

At lower neutron energies (0.1–1 MeV) (Figure 12a–c), the distributions are characterized by numerous localized clusters of interaction events distributed around the primary beam axis. These clusters correspond to recoil nuclei and low-energy reaction products, which deposit their energy over relatively short distances. As a result, the spatial pattern appears fragmented and strongly nonuniform, with pronounced event-to-event fluctuations.

At intermediate energies (around 2 MeV) (Figure 12d), the density of secondary events remains relatively high, and the spatial distribution extends further from the beam axis, indicating an increase in the range and energy of secondary particles.

A notable change is observed at 5 MeV (Figure 12e), where the overall density of secondary interaction events decreases significantly. The spatial distribution becomes more sparse, and the number of pronounced clusters is reduced. This behavior suggests that, despite the higher incident energy, the efficiency of local energy transfer via secondary particles is reduced, likely due to an increased neutron mean free path and a lower probability of interactions within the finite thickness of the absorber. At 14 MeV (Figure 12f), the spatial structure becomes more heterogeneous again, with distinct but relatively isolated clusters of interaction events distributed along and around the beam direction.

This reflects the activation of additional reaction channels and the production of higher-energy secondary particles, which can travel larger distances and deposit energy in more spatially separated regions.

Overall, the secondary-particle maps demonstrate that the spatial characteristics of neutron-induced energy transfer are strongly non-monotonic with respect to incident energy.

The most pronounced and spatially extended interaction patterns are observed in the low- to intermediate-energy range, while higher energies lead to a sparser and more distributed event topology.

**Figure 13:**
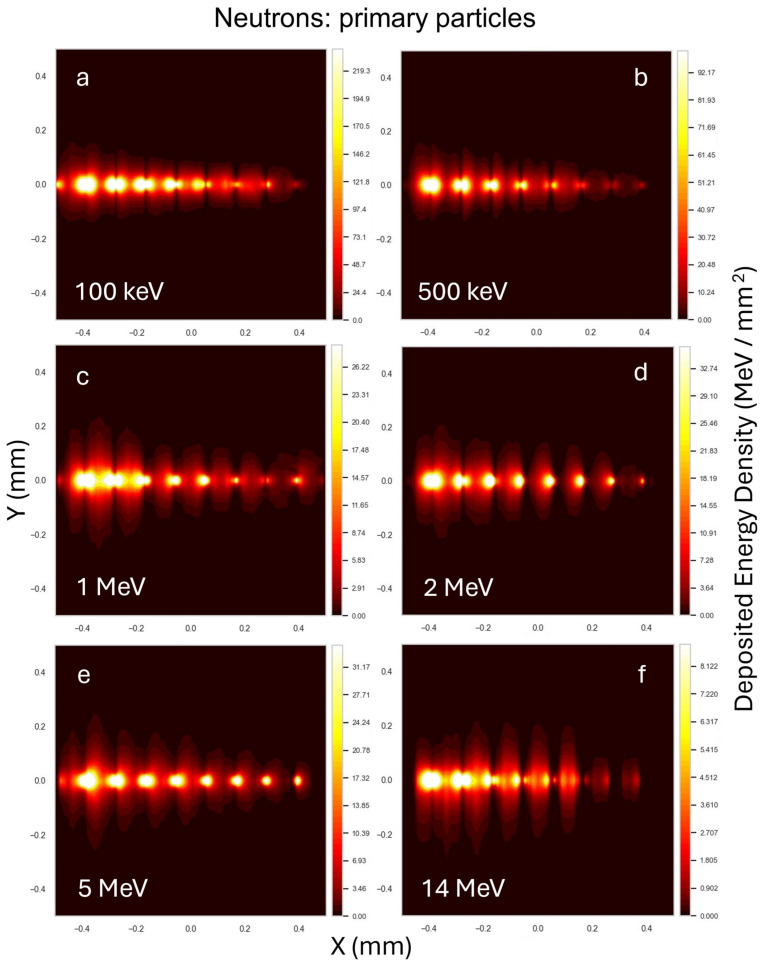
Heat maps of released energy associated with primary neutrons in a 1 mm thick MAPbI_3_ layer for incident energies of (**a**) 0.1 MeV, (**b**) 0.5 MeV, (**c**) 1 MeV, (**d**) 2 MeV, (**e**) 5 MeV, (**f**) 14 MeV. The maximum color scale values are approximately 219.3, 92.17, 26.22, 32.74, 31.17, and 8.12 MeV/mm^2^, respectively.

The spatial distribution of deposited energy associated with primary neutron interactions is shown in Figure 13. In contrast to electrons and photons, neutrons do not lose energy continuously along their trajectories. Energy transfer occurs only through discrete nuclear interactions, which produces a sparse and discontinuous deposition pattern.

The maximum deposited energy density decreases from about 219.3 at 0.1 MeV to 8.12 at 14 MeV, although small local increases are observed at 2 and 5 MeV. This trend indicates that direct energy transfer by primary neutrons becomes less efficient as the neutron energy increases. At higher energies, a larger fraction of neutrons can pass through the material or transfer energy to secondary particles rather than depositing it directly along the primary path.

The maps in Figure 13 also show that the primary neutron contribution remains concentrated near isolated interaction sites. At 0.1 and 0.5 MeV, the local maxima are stronger because neutron scattering can transfer a noticeable fraction of energy within a limited region. At 1 to 5 MeV, the deposition becomes more fragmented along the beam direction. At 14 MeV, the direct primary contribution is weakest on the plotted scale, which reflects the reduced local stopping efficiency of primary neutrons in the 1 mm layer.

**Figure 14:**
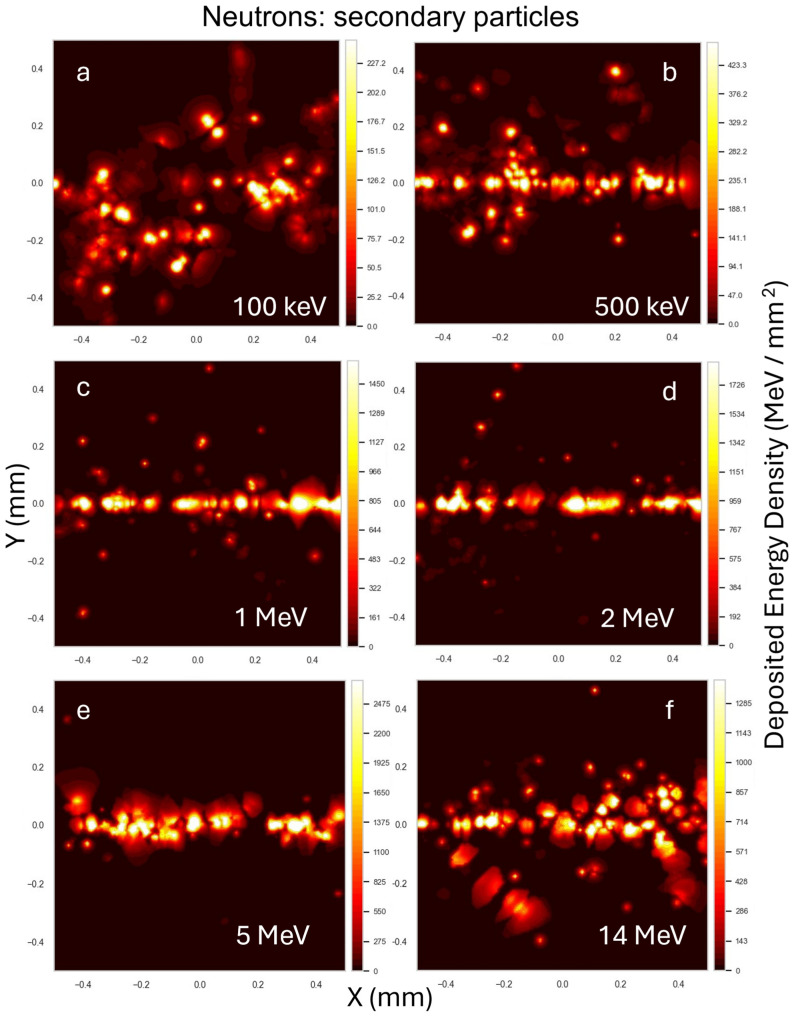
Heat maps of released energy associated with secondary particles in a 1 mm thick MAPbI_3_ layer for incident energies of (**a**) 0.1 MeV, (**b**) 0.5 MeV, (**c**) 1 MeV, (**d**) 2 MeV, (**e**) 5 MeV, (**f**) 14 MeV. The maximum color scale values are approximately 227.2, 423.3, 1450, 1726, 2475, and 1285 MeV/mm^2^, respectively.

The deposited energy associated with secondary particles is shown in Figure 14. These maps exhibit much higher absolute scale values than the primary neutron maps, with maxima of about 227.2, 423.3, 1450, 1726, 2475, and 1285 MeV/mm^2^ for increasing neutron energy. This confirms that the main energy release under neutron irradiation is produced by secondary particles rather than by primary neutrons. The increase from 0.1 MeV to 5 MeV reflects the growing role of recoil particles and nuclear reaction products, which can deposit large amounts of energy locally.

At 14 MeV, the maximum value decreases compared with 5 MeV, but the spatial distribution remains highly heterogeneous and contains pronounced hotspots. This behavior is consistent with the stochastic nature of neutron interactions. Higher energy neutrons can generate more energetic secondary particles, but part of this energy may be transported over longer distances or leave the sensitive volume before being fully deposited. Therefore, the secondary particle response is not simply proportional to incident neutron energy. It is controlled by the balance between interaction probability, secondary particle range, and local energy deposition efficiency. Overall, Figure 13 and Figure 14 show that neutron irradiation produces weak direct energy deposition by primary neutrons and a much stronger, localized response from secondary charged particles.

Representative 3D particle trajectories under neutron irradiation are shown in Figure S8.

Overall, the released energy associated with secondary particles demonstrates a strongly non-monotonic dependence on neutron energy. While low and intermediate energies favor more localized and volumetrically distributed energy transfer, higher energies lead to a more sparse but spatially extended pattern with pronounced local hotspots.

**Figure 15:**
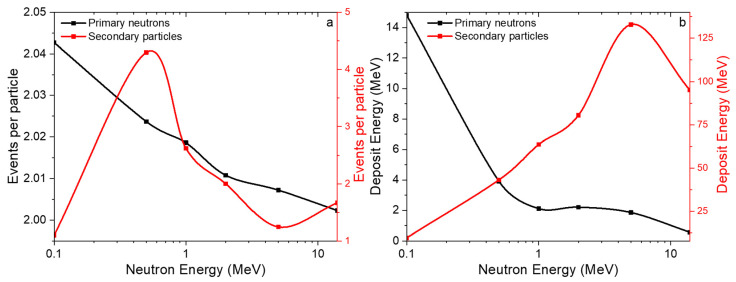
Integral characteristics of neutron–perovskite interaction: (**a**) events per particle, (**b**) deposited energy.

The integral characteristics of neutron interaction in MAPbI_3_ are summarized in Figure 15. For primary neutrons, the number of recorded events remains close to a nearly constant level over the considered energy range, with only a slight decrease at higher energies. This behavior reflects the neutral nature of neutron transport: neutrons do not lose energy continuously and interact only through discrete nuclear collisions. The direct energy release by primary neutrons decreases with increasing energy, indicating that the primary neutron contribution becomes less efficient within the finite 1 mm absorber as the neutron mean free path increases.

The secondary particle contribution shows a much stronger energy dependence. The number of secondary events reaches a maximum in the low-energy range and then decreases toward higher energies. This indicates that the probability of producing secondary particles inside the absorber is highest when neutron interaction probability and energy transfer efficiency are balanced. At higher neutron energies, fewer interactions occur inside the finite material volume, although additional reaction channels may become available.

The deposited energy associated with secondary particles follows a different trend.

It increases with neutron energy and reaches a maximum around 5 MeV, after which it decreases at 14 MeV. This behavior is physically important: the highest incident neutron energy does not necessarily produce the strongest local response. At intermediate energies, secondary particles are sufficiently energetic and still produced with enough probability to deposit a large fraction of their energy inside the absorber. At 14 MeV, part of the energy can be carried by more penetrating reaction products or transported beyond the sensitive volume before being deposited. As a result, the local deposited energy decreases despite the higher incident neutron energy.

Overall, Figure 15 confirms that neutron-induced energy deposition in MAPbI_3_ is controlled mainly by secondary charged particles rather than by direct neutron energy loss. The most efficient energy transfer occurs in the intermediate-energy range, where the balance between interaction probability, secondary particle production, and local stopping is most favorable.

Additional process-specific integral characteristics for neutron irradiation are provided in Supplementary Information (Figures S9 and S10). Only integral process contributions are included there; process-specific spatial maps are not presented, since the present work focuses on total primary and secondary particle response.

### 3.4. Multilayer Detector

While the results presented above provide a detailed understanding of radiation–matter interaction mechanisms in a single-layer MAPbI_3_ system, real devices are typically characterized by more complex, multilayer architectures. In practical applications such as radiation detectors, imaging systems, or energy-related devices, perovskite layers are combined with transport layers, electrodes, or other functional materials, forming structures with spatially varying composition and thickness.

In such systems, the spatial distribution of interaction events and energy deposition may be significantly modified by interfaces, material contrasts, and layer-specific transport properties. In particular, the propagation of secondary particles across layer boundaries, as well as the redistribution of deposited energy between different functional regions, can play a critical role in determining the overall device response.

To assess these effects and to bridge the gap between idealized material-level modeling and realistic device configurations, an additional set of simulations was performed for a simplified multilayer perovskite-based structure. This allows us to examine how the interaction patterns identified in the single-layer model are modified in the presence of layered geometries and to evaluate the potential implications for detector performance and radiation response in practical systems.

For the multilayer detector geometries, the analysis was focused on how the surrounding functional layers modify the spatial response of the MAPbI_3_ absorber under representative photon, electron, and neutron irradiation. In contrast to the single-layer calculations discussed above, these structures include thin transport layers and electrodes together with a comparatively thick perovskite region. Therefore, the spatial maps should not be interpreted only as intrinsic MAPbI_3_ response, but rather as the response of the complete detector-like stack.

Because the layer thicknesses differ by several orders of magnitude, the thick perovskite absorber naturally dominates the visible spatial distributions, while ultrathin contacts and transport layers appear mainly as narrow interface regions. For this reason, the following discussion considers not only where the interaction density is highest, but also whether the response is governed by primary-particle transport or by secondary-particle generation. This distinction is especially important for comparing photons, electrons, and neutrons, since these projectiles transfer energy through fundamentally different mechanisms.

**Figure 16:**
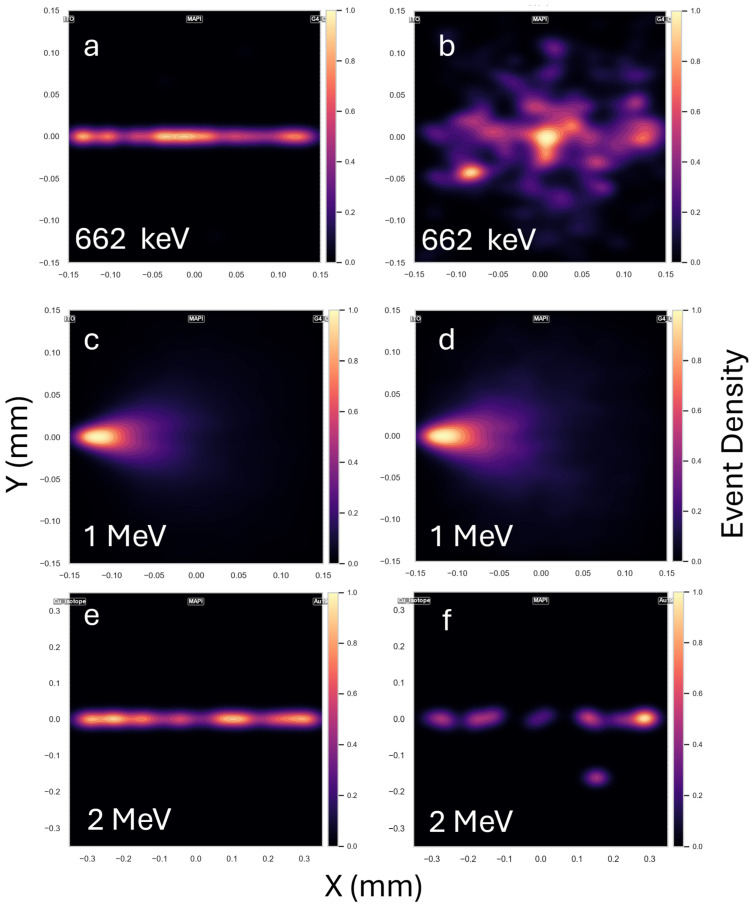
Event-density maps in multilayer MAPbI_3_-based detector structures under irradiation by 662 keV photons, 1 MeV electrons, and 2 MeV neutrons: contributions from primary particles (**a**,**c**,**e**) and secondary particles (**b**,**d**,**f**). Rows correspond to (**a**,**b**) photons, (**c**,**d**) electrons, and (**e**,**f**) neutrons.

The event-density maps are first used to identify the regions where particle interactions occur within the multilayer structures (Figure 16). The multilayer detector simulations reveal substantial differences in the spatial distribution of interaction events depending on the type of incident radiation. For primary photons (Figure 16a), the event density is concentrated in a narrow axial region extending through the detector stack.

This behavior reflects the weakly interacting nature of gamma quanta: they do not lose energy continuously but instead propagate over relatively long distances and interact only at discrete points through processes such as Compton scattering and the photoelectric effect. As a result, the primary-photon event pattern remains highly collimated and does not exhibit strong lateral spreading.

In contrast, the events associated with secondary particles produced by photons (Figure 16b) are distributed over a much broader volume of the detector. This broader spatial pattern is physically expected, since the main consequence of photon interaction in the MAPbI_3_-based structure is the generation of secondary charged particles, primarily Compton electrons, photoelectrons, and low-energy de-excitation products such as characteristic X-rays and Auger electrons. Once created, these secondaries undergo multiple scattering and deposit energy over a wider region than the primary gamma quanta themselves. Therefore, even though the primary-photon map is narrow and track-like, the secondary-particle map becomes diffuse, spatially extended, and locally heterogeneous.

For electron irradiation, the event-density maps exhibit a different morphology. The map for primary electrons (Figure 16c) shows a pronounced forward-directed conical distribution beginning at the entrance side of the detector. This pattern is characteristic of charged-particle transport in matter: electrons interact continuously through ionization and excitation, while multiple Coulomb scattering progressively broadens the beam as it penetrates the absorber.

Accordingly, the highest event density is observed near the entrance region, after which the distribution expands laterally and gradually decreases in intensity with depth. The corresponding map for secondary particles (Figure 16d) remains forward-oriented but is broader and more diffuse. This behavior reflects the generation of secondary electrons along the primary-electron track, which redistribute interactions over a wider effective volume. Thus, in the electron case, both primary and secondary contributions are spatially extended, but the primary electrons preserve the main transport direction, whereas the secondaries enhance volumetric spreading.

**Figure 17:**
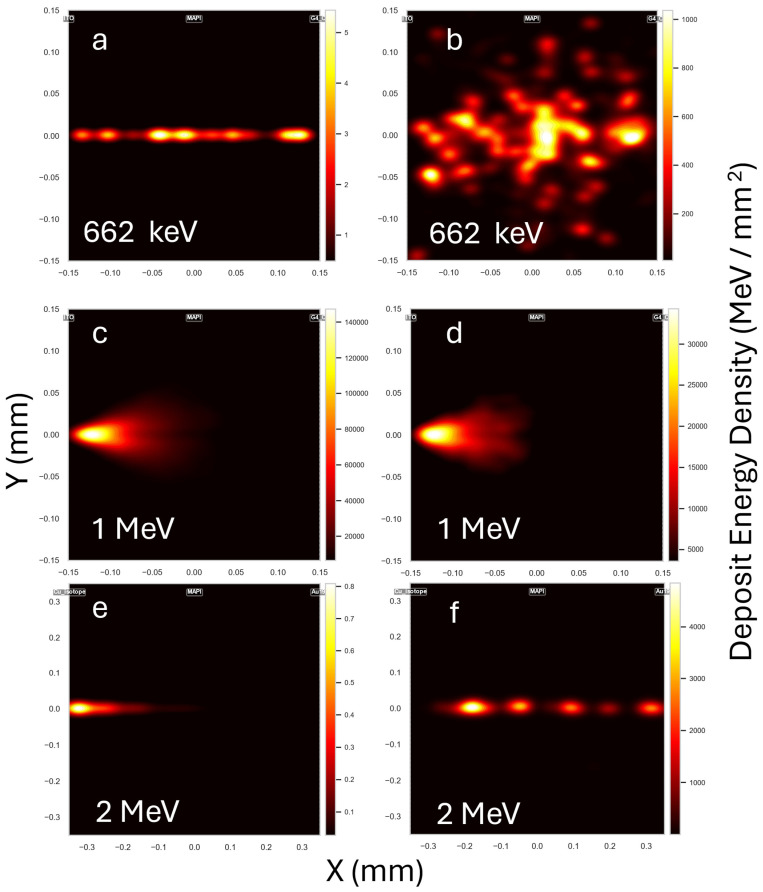
Maps of released energy in multilayer MAPbI_3_-based detector structures under irradiation by 662 keV photons, 1 MeV electrons, and 2 MeV neutrons: contributions from primary particles (**a**,**c**,**e**) and secondary particles (**b**,**d**,**f**). Rows correspond to (**a**,**b**) photons, (**c**,**d**) electrons, and (**e**,**f**) neutrons.

The neutron-induced event-density maps differ qualitatively from both the photon and electron cases. For primary neutrons (Figure 16e), the events are sparse and arranged mainly along the beam axis, indicating that neutrons traverse the detector without continuous ionization loss and interact only through discrete nuclear collisions. Because neutron transport is governed by elastic and inelastic nuclear interactions rather than electromagnetic stopping, the resulting primary-event pattern is weakly structured and spatially discontinuous. The secondary event map (Figure 16f) is more heterogeneous and consists of several localized clusters. These clusters are associated with neutron-induced reaction products and recoil particles generated in the multilayer structure. In this case, the observable detector response is produced predominantly by secondary charged particles rather than by the neutrons themselves. The fragmented topology of the secondary map reflects the stochastic character of neutron interactions and the localized nature of energy transfer through recoil nuclei and other secondary products. The corresponding energy-release maps (Figure 17) are then analyzed to determine whether these interactions produce significant local energy deposition.

The spatial maps of released energy further clarify how the deposited energy is redistributed inside the multilayer detector for different types of incident radiation. For primary photons (Figure 17a), the released energy is confined to a narrow region along the beam direction, closely following the spatial pattern already observed for primary-photon interaction events. This reflects the fact that gamma quanta themselves transfer energy only when a discrete interaction occurs. Since the number of such direct interactions is limited, the energy released by primary photons remains relatively localized and does not form a broad volumetric distribution.

A markedly different behavior is observed for the photon-induced secondary particles (Figure 17b). Here the energy release is distributed over a much broader region and exhibits multiple localized hotspots. This result is physically consistent with the dominant role of secondary electrons in gamma detection. At 662 keV, the absorbed photon energy is transferred mainly to Compton electrons and, to a lesser extent, photoelectrons and atomic de-excitation products. These charged secondaries deposit energy much more efficiently than the primary photons, producing a spatially extended and heterogeneous energy-release map. Therefore, in the photon case, the main contribution to detector response arises not from the direct action of the incident gamma quanta, but from the secondary electron cascade they generate in the multilayer MAPbI_3_ structure.

For electron irradiation, the energy-release maps are dominated by the primary electrons. In Figure 17c, the deposited energy forms a pronounced forward-oriented conical region with the maximum concentrated near the entrance side of the detector. This morphology is a direct consequence of continuous ionization losses and repeated multiple scattering. As electrons enter the active structure, they immediately begin to lose energy, and a substantial fraction of that energy is deposited in the near-entrance part of the MAPbI_3_-containing stack. With increasing penetration depth, lateral spreading becomes more pronounced, and the energy density decreases, forming the characteristic tapered profile visible in the map.

The corresponding secondary-particle contribution (Figure 17d) follows a similar overall geometry but is more diffuse and less intense. This indicates that secondary electrons and other accompanying products broaden the effective deposition region, yet the dominant part of the energy transfer is still governed by the primary electrons themselves. Hence, unlike the photon case, the electron response is controlled primarily by direct transport of the incident particle, with secondaries acting mainly as a redistribution mechanism.

For neutrons, the primary-particle energy-release map (Figure 17e) is weak and highly localized, indicating that the neutrons themselves contribute only modestly to direct energy deposition. This is expected because neutrons are electrically neutral and do not ionize matter continuously.

Instead, they transfer energy only through nuclear collisions, and the corresponding direct energy deposition by the primary neutron remains small and spatially discrete. In contrast, the energy released by secondary particles (Figure 17f) is substantially more pronounced and appears as a sequence of localized hotspots along the detector. These hotspots reflect the production of recoil particles and other neutron-induced secondaries, which deposit their energy over short distances and therefore create strongly localized maxima. The spatially discontinuous nature of the pattern again emphasizes that neutron response in the detector is indirect and is mediated by secondary-particle generation rather than by direct energy loss of the primary neutron.

Overall, Figure 16 demonstrates that the event topology in multilayer MAPbI_3_-based detectors is strongly radiation-type-dependent. Photon primaries remain weakly interacting and highly collimated, but their secondaries spread over a much larger volume. Electrons produce the most continuous and forward-developed interaction region because they directly ionize the material along their trajectory. Neutrons, by contrast, generate a sparse primary-event pattern, while the detectable response emerges mainly through spatially separated clusters of secondary-particle interactions. Thus, the multilayer detector response is controlled not only by the absorber material itself, but also by the fundamental difference between direct charged-particle transport and indirect energy transfer mediated by secondary cascades.

The maps in Figure 17 show that the balance between primary and secondary contributions to energy deposition depends strongly on the radiation type. In the photon case, secondary particles dominate the released-energy distribution, confirming that gamma detection in APbI_3_-based multilayer structures is fundamentally secondary-driven. In the electron case, the primary electrons are responsible for the main part of the deposited energy, while secondaries mainly increase the spatial extent of the deposition region. In the neutron case, the opposite extreme is observed: the direct contribution of primary neutrons is weak, and the detector response is determined mainly by localized energy release from neutron-induced secondary particles. Thus, the energy-release maps consistently demonstrate that the physical origin of detector response changes from direct charged-particle stopping for electrons to secondary-mediated energy transfer for photons and neutrons.

After the spatial maps, the multilayer-detector response was further analyzed using integral interaction characteristics (Tables S1 and S2). While Figure 16 and Figure 17 illustrate the spatial topology of particle interactions and energy release, they do not directly quantify the relative contribution of primary and secondary particles. Therefore, the map-based analysis was complemented by tabulated values of the number of recorded events and the corresponding released energy. These quantities make it possible to distinguish between cases where a particle type produces many low-energy interaction steps and cases where rare interactions lead to relatively large local energy release. This distinction is especially important for comparing photons, electrons, and neutrons, because their detector response is governed by different physical mechanisms.

The tabulated values (Table S1) confirm that photon irradiation is governed mainly by secondary-particle production. The direct contribution of primary photons is very small, whereas photon-induced secondary particles dominate both the number of recorded events and the released energy. This reflects the indirect nature of gamma interaction: primary photons transfer energy only through discrete interaction events, after which secondary electrons and de-excitation products determine the local energy deposition. For electron irradiation, the response is dominated by primary particles. Secondary cascades are also substantial, but the main energy transfer remains controlled by direct electron slowing down through ionization, scattering, and radiative losses. This is consistent with the continuous and forward-directed energy-release pattern observed in the spatial maps. For neutron irradiation, both primary and secondary event counts remain low, but the energy balance is controlled by secondary particles. The neutron response is therefore sparse but locally intense: the main contribution comes from recoil particles and other nuclear-reaction products rather than from direct energy loss of the primary neutrons.

Overall, Table S1 supports the trends observed in the maps. Photon response is secondary-mediated, electron response is mainly controlled by direct charged-particle stopping, and neutron response is dominated by rare but energetic secondary-particle events.

After the integral comparison, a process-resolved analysis was performed to identify which interaction channels are responsible for the recorded events and energy release. This additional breakdown is important because the total primary and secondary contributions do not show whether the response is controlled by photon interactions, electron ionization, multiple scattering, bremsstrahlung, or neutron-induced nuclear processes. The corresponding data are summarized in Table S2.

The process-resolved data show that the photon case is initiated mainly by rare Compton and photoelectric interactions of the primary gamma quanta, while Rayleigh scattering contributes to the event count without direct energy release. The much larger secondary-particle contribution is associated mainly with electron ionization and multiple-scattering-limited transport steps, confirming that the detector response to 662 keV photons is governed by secondary electrons rather than by direct photon energy deposition.

For electron irradiation, the table shows a dense charged-particle transport regime. Primary electrons generate a large number of transport steps, with energy release accumulated mainly during multiple-scattering- and ionization-associated steps, while bremsstrahlung provides an additional but smaller contribution. Secondary particles follow the same general pattern: ionization and multiple scattering dominate the event statistics, whereas bremsstrahlung photons and subsequent photon interactions provide smaller corrections. This supports the conclusion that the electron response is primarily controlled by direct charged particle slowing down and the secondary electron cascade.

For neutron irradiation, the process structure is qualitatively different. Primary neutrons interact only through rare hadronic elastic and inelastic events and release very little energy directly.

The dominant energy release appears in the secondary-particle block, especially through hadronic ionization and ion ionization. This indicates that the neutron response is produced mainly by charged recoil particles and nuclear-reaction products generated after neutron interactions. Thus, Table S2 confirms the sparse but locally intense character of neutron-induced energy deposition observed in the spatial maps.

It should also be noted that the process label in the table corresponds to the process assigned to the recorded step. Therefore, energy listed under multiple scattering should be interpreted as energy deposited during steps limited or classified by multiple scattering, rather than as energy deposited by scattering itself. This is particularly relevant for electron transport, where ionization energy loss is accumulated along many transport steps.

Overall, the simulations show that the radiation response of MAPbI_3_ is not determined only by the total amount of released energy, but also by the way this energy is spatially distributed and by whether it is delivered by primary or secondary particles. Electrons, photons, and neutrons produce qualitatively different interaction patterns, which implies different possible damage mechanisms in perovskite-based devices.

For electron irradiation, MAPbI_3_ exhibits the most continuous and spatially extended response. Primary electrons lose energy directly through charged-particle stopping, while secondary electrons broaden the affected volume and introduce additional local heterogeneity. At low energy, the response is concentrated near the entrance surface, whereas at MeV energies it becomes forward-directed and penetrates deeply into the absorber. From the point of view of possible radiation effects, these results indicate where local excitation and energy release may be concentrated. However, the present simulations do not directly predict defect formation, ion migration, or changes in device performance. For detector applications, the same feature is beneficial because charged particles can generate a strong and spatially continuous signal within the active perovskite layer.

Photon irradiation shows a different behavior. Primary gamma quanta interact only weakly and mainly act as initiators of secondary-particle cascades. The energy deposition is therefore governed not by direct photon stopping, but by Compton electrons, photoelectrons, and atomic de-excitation products. This explains why the primary-photon maps remain narrow and sparse, while the secondary-particle maps define the actual energy-release morphology. For MAPbI_3_-based X-ray and gamma detectors, this confirms that the detector response should be interpreted primarily as a secondary-electron-mediated process. From a transport perspective, the most important regions are not necessarily the photon trajectories themselves, but the localized zones where secondary electrons deposit energy.

Neutron irradiation produces the most discontinuous and localized response. Since neutrons do not ionize matter continuously, their direct energy release in MAPbI_3_ is weak. The dominant contribution comes from secondary charged particles generated in elastic and inelastic nuclear interactions. These events are relatively rare, but they can release large amounts of energy locally through recoil particles, hadronic ionization, and ion ionization. As a result, neutron-induced damage is expected to be more stochastic and hotspot-like than electron- or photon-induced damage. This may be relevant for radiation-stability assessment, because a small number of nuclear events can create strongly localized regions of structural disturbance. However, direct defect formation was not evaluated in the present work.

The multilayer detector simulations further show that realistic device stacks preserve the main physical trends observed in the single-layer MAPbI_3_ model, while adding the influence of interfaces and functional layers. The thick perovskite absorber dominates the visible spatial response, but thin transport layers and electrodes can still affect the entrance conditions, secondary-particle generation, and layer-resolved energy deposition.

Taken together, these results indicate that MAPbI_3_-based structures can respond to different radiation fields through distinct mechanisms: direct charged-particle stopping for electrons, secondary-electron cascades for photons, and localized nuclear-reaction products for neutrons. This distinction is important for the design of perovskite radiation detectors, imaging structures, and radiation-tolerant optoelectronic devices. It also suggests that mitigation strategies should be radiation-specific: electron exposure requires control of charged-particle stopping and interface losses, photon detection depends on efficient collection of secondary-electron-generated charge, and neutron environments require attention to rare but highly localized nuclear energy release events.

## 4. Conclusions

In this work, Geant4 simulations were used to analyze radiation interaction in MAPbI_3_ with emphasis on spatially resolved energy release, event topology, and process-dependent transport behavior. The results demonstrate that the radiation response of this material cannot be described only by integral deposited-energy values. The spatial morphology of energy deposition, the role of secondary particles, and the dominant interaction channels are equally important for interpreting both radiation sensitivity and possible radiation-related effects in perovskite-based structures.

The comparison of electrons, photons, and neutrons showed that each type of incident radiation produces a distinct response mechanism. Electron irradiation is mainly associated with direct charged-particle stopping and extended transport through the absorber. Photon irradiation is governed primarily by secondary charged particles generated after relatively rare primary photon interactions. Neutron irradiation produces the most discontinuous response, where direct neutron energy loss is weak and the main local energy release is associated with secondary particles generated in nuclear interactions. These differences indicate that radiation effects in MAPbI_3_ are strongly particle-specific and that the same material may exhibit substantially different local excitation or energy deposition patterns depending on the irradiation environment.

The multilayer simulations further showed that the main interaction mechanisms identified in the single-layer model are preserved in detector-like MAPbI_3_-containing stacks, but the presence of contacts, transport layers, interfaces, and different absorber thicknesses modifies the spatial distribution of events and energy release. This is important for practical detector structures, where possible radiation-induced degradation pathways are not determined only by the active perovskite layer, but also by the redistribution of secondary particles across the full device geometry.

From an application perspective, the results suggest that different radiation fields require different design considerations. For electron exposure, the key issue is the control of charged particle stopping and energy loss near interfaces or within the active layer. For photon detection, efficient collection of charge generated by secondary electrons is critical. For neutron environments, attention should be paid to localized high-energy secondary products, which may produce sparse but intense regions of structural disturbance. Thus, radiation-response optimization in MAPbI_3_-based devices should be specific to the intended radiation type rather than based only on general material composition.

At the same time, the present study has several limitations. The single-layer 1 mm MAPbI_3_ geometry was intentionally selected to improve interaction statistics and to reveal material-level transport regimes. Consequently, the obtained interaction probabilities, deposited energy distributions, and hotspot intensities should not be interpreted as direct predictions for typical submicron perovskite solar cell absorbers. Instead, the single-layer model is used here to identify particle-dependent transport mechanisms and spatial energy deposition morphologies. The irradiation source was modeled as point-like and monoenergetic, whereas realistic conditions may involve extended beams, angular distributions, energy spectra, pulsed irradiation, or more complex source laws. Future simulations should therefore include different irradiation geometries and source distributions to assess how beam shape and spectral composition affect the spatial response. In addition, the device models used here represent simplified multilayer geometries. More realistic simulations should include full device architectures, encapsulation, substrates, patterned electrodes, contacts, and possible nonuniformities in the active layer.

Another limitation is related to statistics and data handling. Increasing the number of primary particles would improve the reliability of rare-event analysis, especially for photon and neutron irradiation. However, this would also lead to much larger step-resolved output files and significantly higher computational and post-processing costs.

Because photon and neutron interactions contain a larger rare event component than electron transport, local hotspot intensities are expected to show stronger statistical fluctuations than integral trends. For this reason, spatial maps are interpreted mainly in terms of stable morphological features, such as track-like, cascade-like, or hotspot-like patterns, rather than as exact values of the local maximum.

Additional quantitative descriptors, such as radial spread, penetration depth, and cluster size distributions, could provide a more detailed description of the spatial morphology. In the present work, these quantities were not used as primary metrics, because the main goal was to compare the dominant transport regimes for electrons, photons, and neutrons rather than to build a complete statistical model of each hotspot. The spatial maps were interpreted together with integral event numbers, deposited energy, maximum local energy density, and process-resolved statistics. Exact cluster sizes were not extracted from the rendered maps, since interpolation and smoothing can modify the apparent size and contrast of local hotspots. A dedicated analysis of radial spread, penetration depth, and cluster statistics directly from the original Geant4 step data can be considered in future work.

Previous numerical studies considered proton-induced ionization, displacement, and heating channels in halide perovskites, as well as secondary particle generation under space relevant irradiation conditions [10,57]. In contrast, the present work focuses on spatially resolved Geant4 transport, deposited energy morphology, and the separation of primary and secondary contributions for electron, photon, and neutron irradiation. Therefore, quantities such as displacement damage and non-ionizing energy loss were not treated as primary outputs here.

The present work does not evaluate displacement damage or non-ionizing energy loss. These quantities require a separate damage model, including displacement threshold energies, primary knock-on atom spectra, and a partition between ionizing and non-ionizing energy loss. In hybrid halide perovskites, this issue is further complicated by the soft ionic lattice and by the important role of electronic excitation, local energy release, chemical instability, and ion migration. Therefore, the present results should not be interpreted as direct predictions of displacement damage or defect yields. They provide a transport-level description of where energy is released, and which primary or secondary particles are responsible for that release.

Finally, Geant4 provides information about particle transport and local energy release, but it does not directly predict the resulting atomic-scale defect structure, ion migration, phase instability, or changes in electronic properties. To connect deposited-energy patterns with material degradation mechanisms, future studies should combine Monte Carlo transport with atomistic approaches such as molecular dynamics and density functional theory. Molecular dynamics can be used to follow local structural disorder and defect formation after energy transfer events, while DFT can help evaluate defect energetics, electronic states, and possible changes in carrier transport. Experimental validation is also necessary, including controlled irradiation studies together with structural, spectroscopic, and electrical characterization. Such combined simulation–experiment workflows would provide a more complete understanding of radiation effects in hybrid halide perovskites and support the design of more robust perovskite-based radiation detectors and optoelectronic devices. 

## Data Availability

The raw data supporting the conclusions of this article will be made available by the authors on request.

## Supplementary Materials

The following supporting information can be downloaded at: https://www.mdpi.com/article/10.3390/nano16130803/s1, Figure S1: Schematic detector architectures (not to scale) used in the simulations: (a) photon and electron irradiation cases, (b) neutron irradiation case; Figure S2: Example of 3D primary (a) and secondary (b) particle trajectories under 8.2 MeV electron irradiation; Figure S3: Process-resolved event statistics as a function of incident-electron energy (a,b—for primary particles, c–e—for secondary particles); Figure S4: Process-resolved energy release statistics as a function of incident-electron energy (a,b—for primary particles, c–e—for secondary particles); Figure S5: Example of 3D primary (a) and secondary (b) particle trajectories under 662 keV photon irradiation; Figure S6: Process-resolved event statistics as a function of incident-photon energy (a,b—for primary particles, c–e—for secondary particles); Figure S7: Process-resolved deposit energy statistics as a function of incident-photon energy (a—for primary particles, b–d—for secondary particles); Figure S8: Example of 3D primary (a) and secondary (b) particle trajectories under 1 MeV neutron irradiation; Figure S9: Process-resolved event statistics as a function of incident-neutron energy (a—for primary particles, b–d—for secondary particles); Figure S10: Process-resolved deposit energy statistics as a function of incident-neutron energy (a—for primary particles, b–d—for secondary particles); Table S1: Integral characteristics of particle interactions in the multilayer APbI_3_-containing detector structures under 662 keV photon, 1 MeV electron, and 2 MeV neutron irradiation; Table S2: Process-resolved interaction statistics for primary and secondary particles in the multilayer APbI_3_-containing detector structures under 662 keV photon, 1 MeV electron, and 2 MeV neutron irradiation.
