# Supplementary material for: From Tracks to Hotspots: Particle-Dependent Radiation Energy Deposition in MAPbI3 Perovskite
Source: Nanomaterials (Basel). 2026 Jun 29;16(13):803. doi: 10.3390/nano16130803 (PMC13362700; doi:10.3390/nano16130803)
Supplement: Supplementary file 1 [file nanomaterials-16-00803-s001.zip › nanomaterials-4397047-supplementary.pdf]

From Tracks to Hotspots: Particle-Dependent Radiation Energy Deposition in $MAPbI_3$ Perovskite

Ivan E. Novoselov[1,2], Zhi Xing[3], Huiliang Sun[3,4], Ivan S. Zhidkov[1,2,5]

[1] Institute of Physics and Technology, Ural Federal University, Mira 19 Street, Yekaterinburg 620062, Russia

[2] Federal Research Center for Problems of Chemical Physics and Medicinal Chemistry of Russian Academy of Sciences, Semenov Av., 1, Chernogolovka, 142432, Russia

[3] School of Chemistry and Chemical Engineering, Gannan Normal University, Ganzhou, Jiangxi, 341000, China

[4] Guangdong Engineering Technology Research Center for Photoelectric Sensing Materials & Devices, Guangzhou Key Laboratory of Sensing Materials & Devices, Center for Advanced Analytical Science, School of Chemistry and Chemical Engineering Guangzhou University, Guangzhou, 510006, China

[5] M.N. Mikheev Institute of Metal Physics of Ural Branch of Russian Academy of Sciences, S. Kovalevskoi 18 Street, Yekaterinburg 620108, Russia

Supplementary information

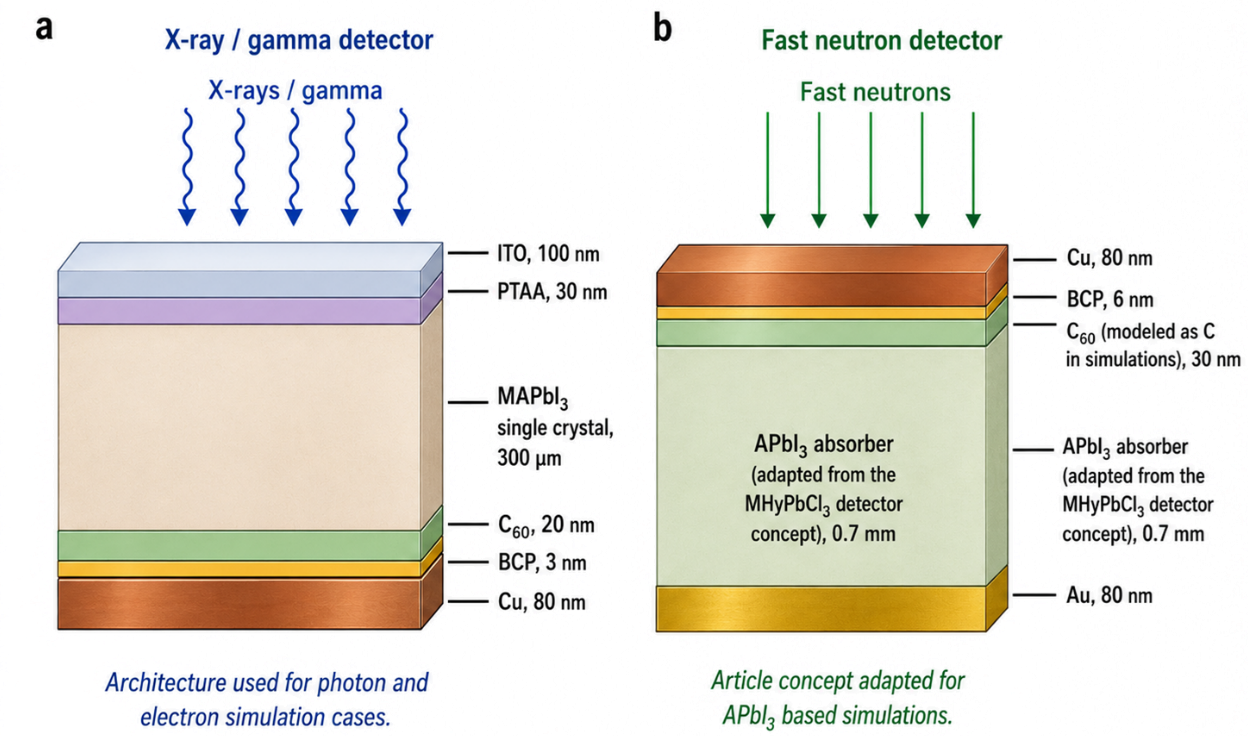

Figure S1. Schematic detector architectures (not to scale) used in the simulations: a – photon and electron irradiation cases, b – neutron irradiation case

For visualization clarity, not all simulated 3D trajectories are shown. Instead, representative trajectories were selected from regions with the highest density of particle steps and interaction events, with preference given to tracks containing the longest continuous segments. This selection was used only for illustrative purposes and did not affect the quantitative analysis.

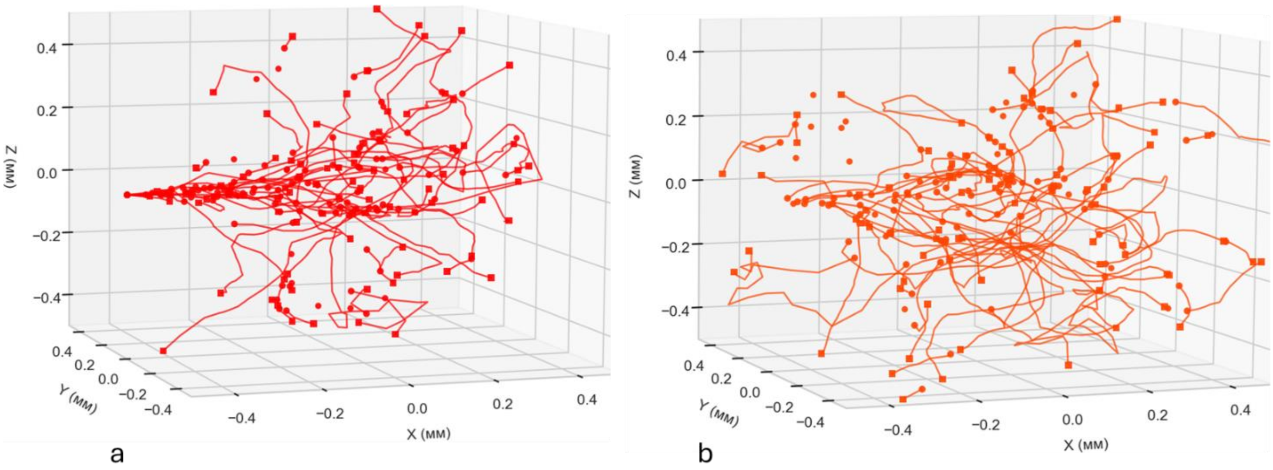

Figure S2. Example of 3D primary (a) and secondary (b) particle trajectories under 8.2 MeV electron irradiation

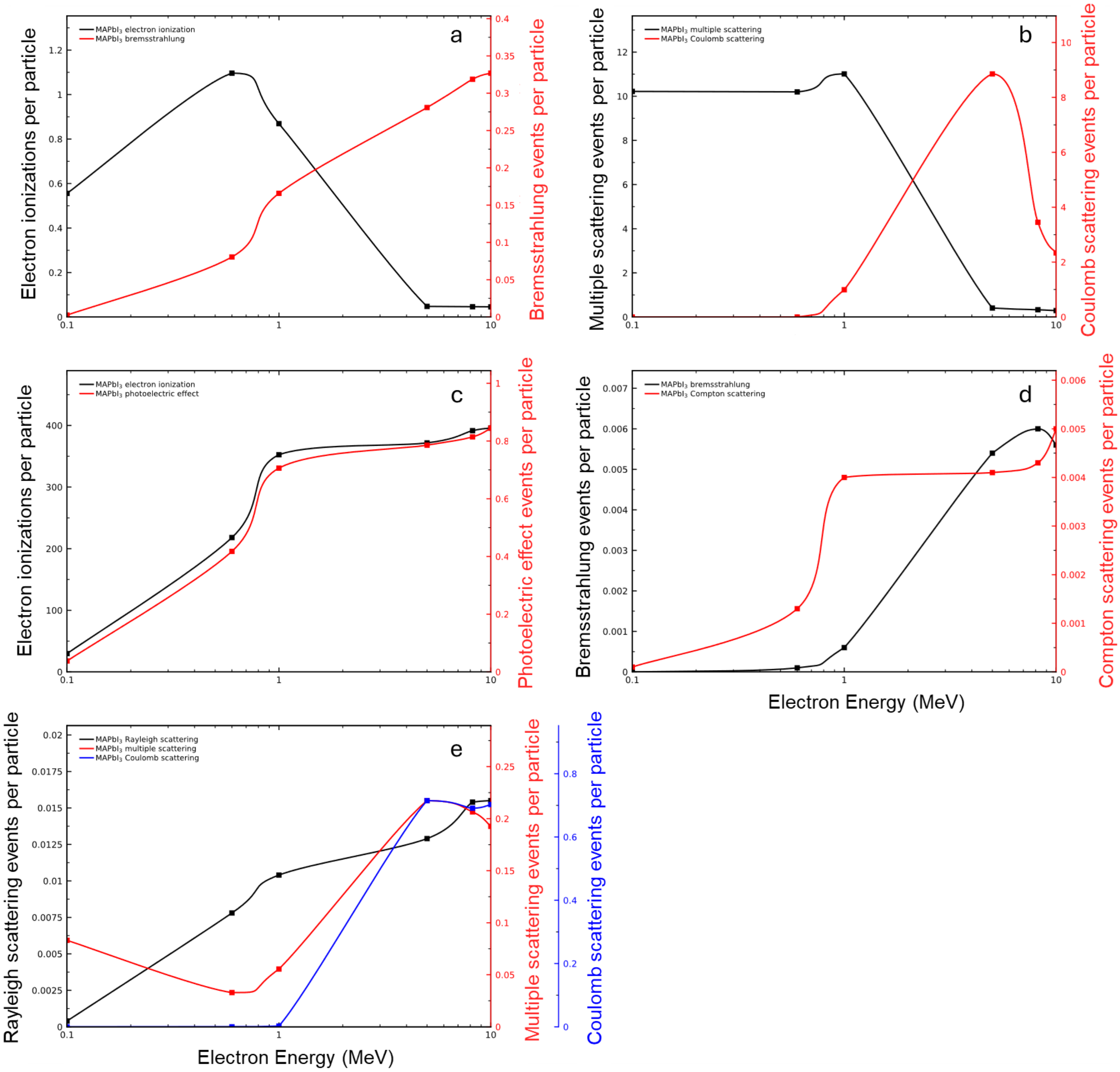

Figure S3. Process-resolved event statistics as a function of incident-electron energy (a, b – for primary particles, c-e – for secondary particles)

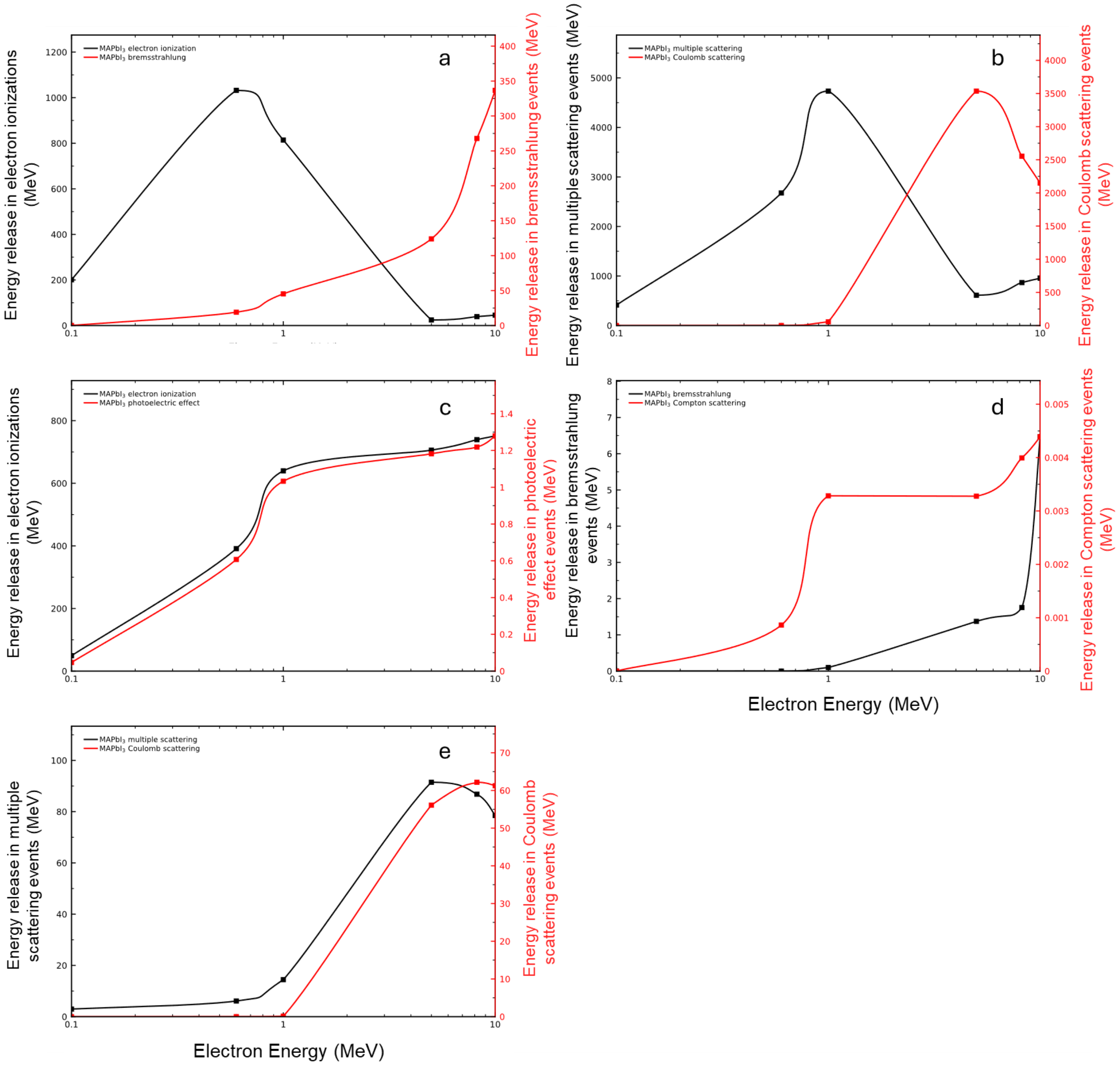

Figure S4. Process-resolved energy release statistics as a function of incident-electron energy (a, b – for primary particles, c-e – for secondary particles)

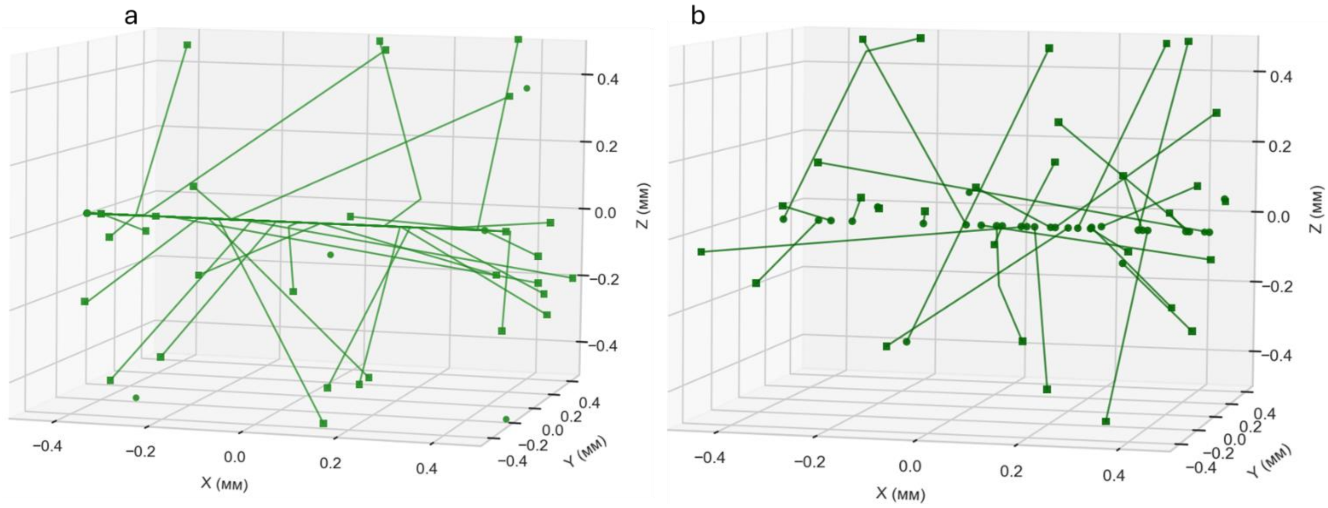

Figure S5. Example of 3D primary (a) and secondary (b) particle trajectories under 662 keV photon irradiation

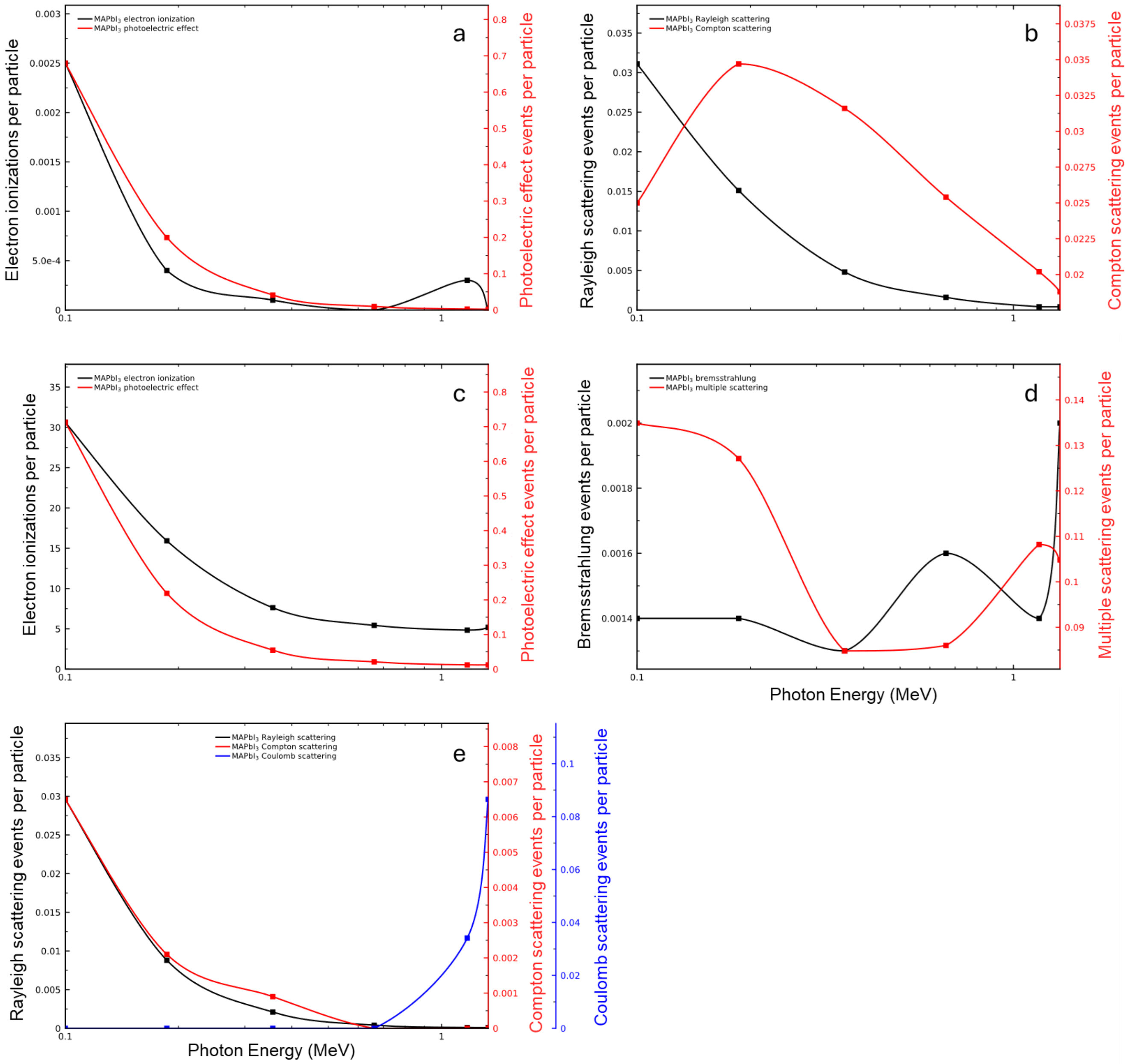

Figure S6. Process-resolved event statistics as a function of incident-photon energy (a, b – for primary particles, c-e – for secondary particles)

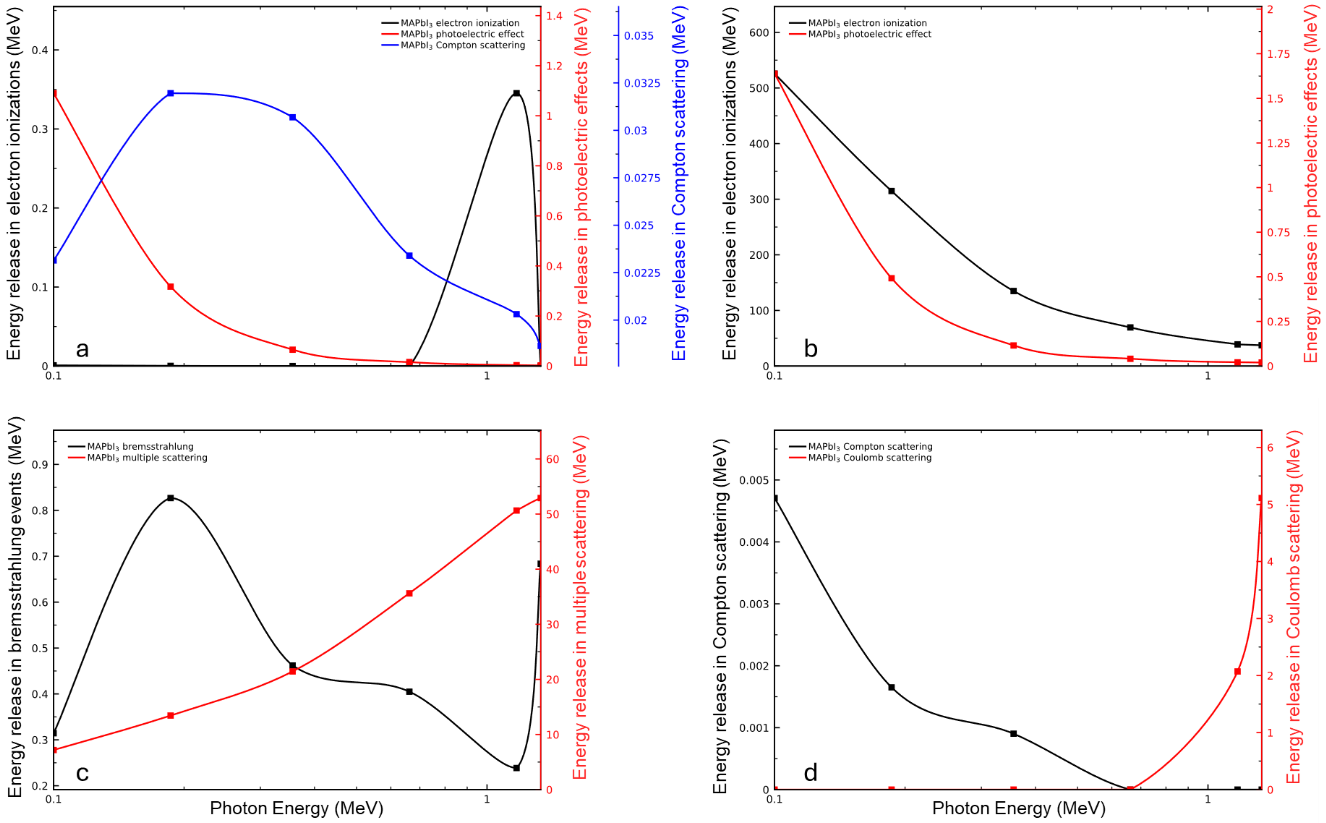

Figure S7. Process-resolved deposit energy statistics as a function of incident-photon energy (a – for primary particles, b-d – for secondary particles)

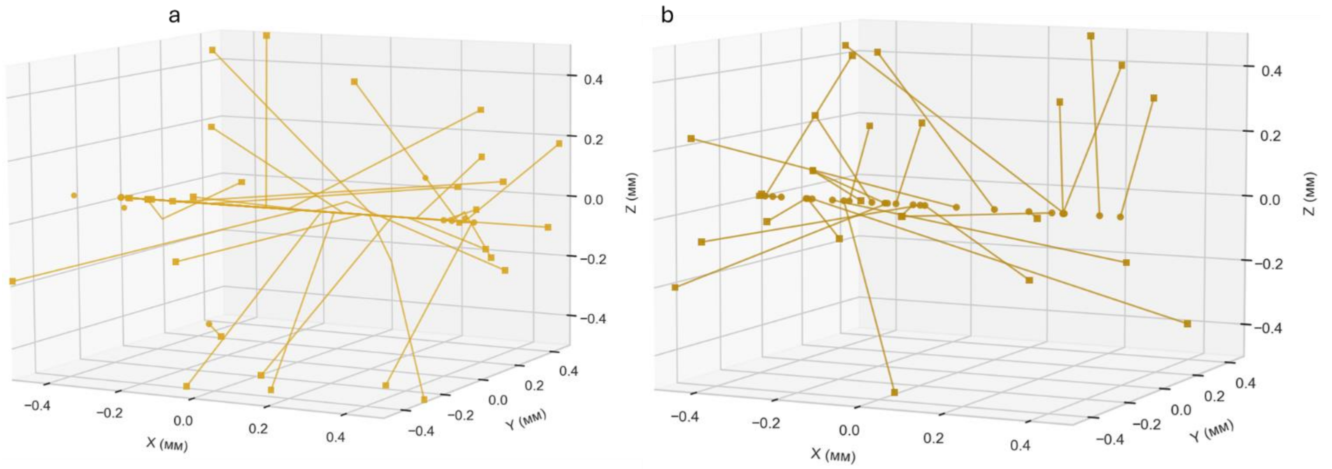

Figure S8. Example of 3D primary (a) and secondary (b) particle trajectories under 1 MeV neutron irradiation

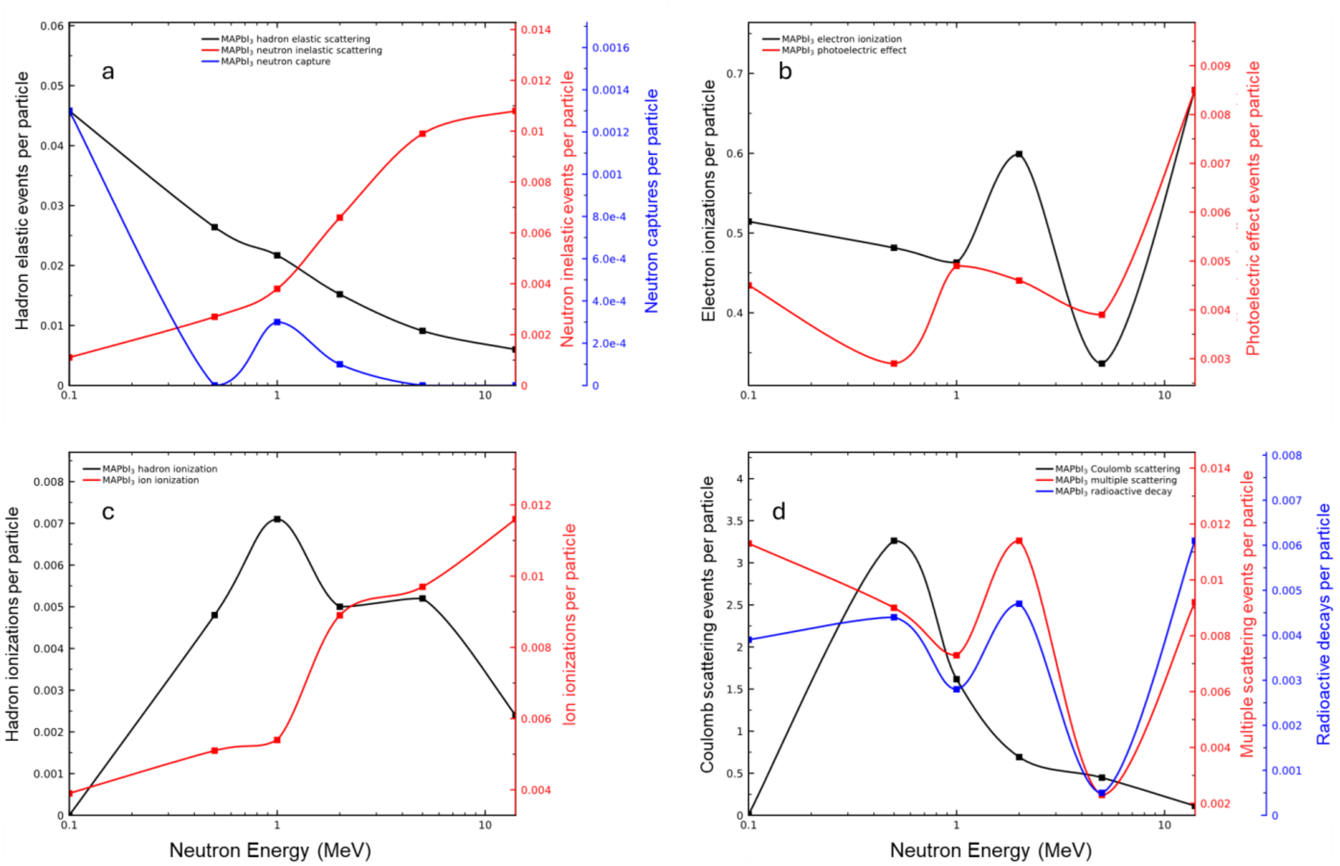

Figure S9. Process-resolved event statistics as a function of incident-neutron energy (a – for primary particles, b-d – for secondary particles)

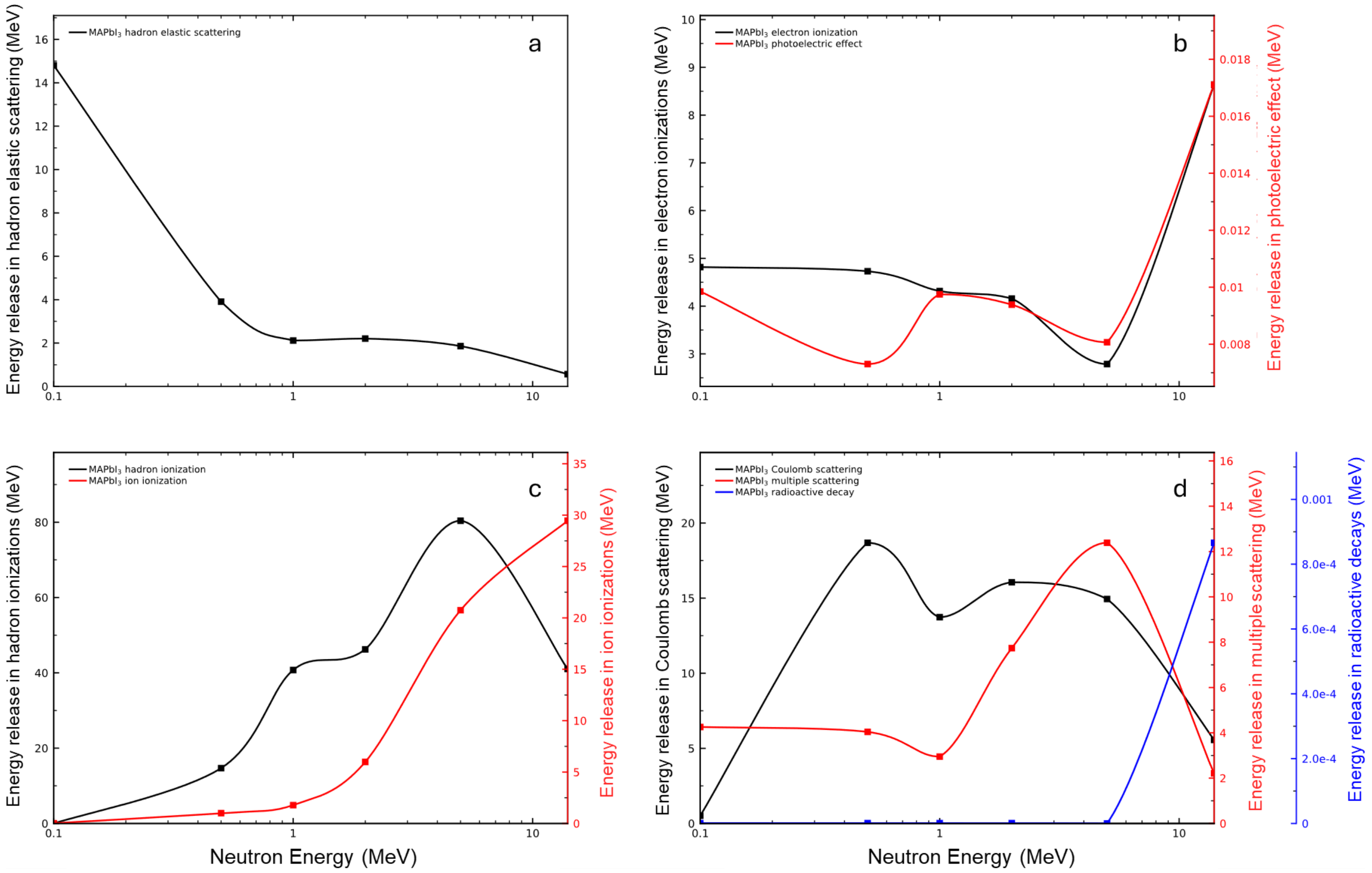

Figure S10. Process-resolved deposit energy statistics as a function of incident-neutron energy (a – for primary particles, b-d – for secondary particles)

Table S1. Integral characteristics of particle interactions in the multilayer $APbI_3$-containing detector structures under 662 keV photon, 1 MeV electron, and 2 MeV neutron irradiation

**Radiation type**	**Parameters**	
	**Primary particles**	
**Photons**	Events per particle	0.0127
	Summary Energy Release, MeV	0.0151
	Mean Energy Release in Particle Step, keV	0.1187
	Max Energy Release in Particle Step, keV	0.7041
	**Secondary particles**	
	Events per particle	3.1584
	Summary Energy Release, MeV	18.8281
	Mean Energy Release in Particle Step, keV	0.5961
	Max Energy Release in Particle Step, MeV	0.0418
**Electrons**	**Primary particles**	
	Events per particle	215.1716
	Summary Energy Release, MeV	1196.4108
	Mean Energy Release in Particle Step, keV	0.5560
	Max Energy Release in Particle Step, MeV	0.0790
	**Secondary particles**	
	Events per particle	158.5982
	Summary Energy Release, MeV	385.8976
	Mean Energy Release in Particle Step, keV	0.2433
	Max Energy Release in Particle Step, MeV	0.0750
**Neutrons**	**Primary particles**	
	Events per particle	0.0143
	Summary Energy Release, keV	3.4921
	Mean Energy Release in Particle Step, keV	0.0244
	Max Energy Release in Particle Step, keV	0.9506
	**Secondary particles**	
	Events per particle	0.0167
	Summary Energy Release, MeV	54.3158
	Mean Energy Release in Particle Step, MeV	0.3252
	Max Energy Release in Particle Step, MeV	1.9530

Table S2. Process-resolved interaction statistics for primary and secondary particles in the multilayer $APbI_3$-containing detector structures under 662 keV photon, 1 MeV electron, and 2 MeV neutron irradiation

Radiation type	Process	Events per particle	Summary Energy Release, MeV	Mean Energy Release in Particle Step, keV	Max Energy Release in Particle Step, MeV
Photons	**Primary particles**				
	Rayleigh Scattering	0.0008	0.0000	0.0000	0.0000
	Compton Scattering	0.0086	0.0079	0.0920	0.0003
	Photoelectric effect	0.0033	0.0072	0.2172	0.0007
	**Secondary particles**				
	Rayleigh Scattering	0.0001	0.0000	0.0000	0.0000
	Bremsstrahlung	0.0014	0.0388	2.7702	0.0173
	Electron Ionization	1.0197	3.0556	0.2997	0.0214
	Multiple Scattering	2.1324	15.7248	0.7374	0.04182
	Photoelectric effect	0.0048	0.0091	0.1885	0.0007
Electrons	**Primary particles**				
	Bremsstrahlung	0.1328	1.7334	1.3052	0.0425
	Electron Ionization	0.2693	4.5457	1.6880	0.0362
	Multiple Scattering	214.7695	1190.1318	0.5541	0.0790
	**Secondary particles**				
	Rayleigh Scattering	0.0031	0.0000	0.0000	0.0000
	Bremsstrahlung	0.0095	0.1878	1.9764	0.0188
	Electron Ionization	94.8100	190.2964	0.2007	0.0395
	Multiple Scattering	63.5073	194.9901	0.3070	0.0750
	Photoelectric effect	0.2681	0.4234	0.1579	0.0008
Neutrons	**Primary particles**				
	Hadron Elastic Scattering	0.0119	0.0035	0.02935	0.0010
	Neutron Inelastic Scattering	0.0024	0.0000	0.0000	0.0000
	**Secondary particles**				
	Electron Ionization	0.0004	0.1029	25.7350	0.0288
	Hadron Ionization	0.0079	51.0126	645.7287	1.9530
	Ion Ionization	0.0078	3.1304	40.1333	0.4582
	Multiple Scattering	0.0003	0.0028	0.9171	0.0014
	Photoelectric effect	0.0003	0.0671	22.3763	0.0332
